# Scalable Coating of Spatially Uniform WBG Perovskites via Inhibiting SAM Aggregation for Flexible All‐Perovskite Tandem Modules

**DOI:** 10.1002/smsc.70379

**Published:** 2026-08-14

**Authors:** Jincheng Luo, Jonathan S. Austin, Alessia Romio, Antonio Cabas Vidani, Huagui Lai, Cédric Bühler, Stefano Saroglia, Pedro Octavio Quintana Ceres, Mirjana Dimitrievska, Thilo Glatzel, Chih‐Jen Shih, Fan Fu

**Affiliations:** ^1^ Laboratory for Thin Films and Photovoltaics Empa ‐ Swiss Federal Laboratories for Materials Science and Technology Dübendorf Switzerland; ^2^ Fluxim AG Winterthur Switzerland; ^3^ Department of Physics University of Basel Basel Switzerland; ^4^ Transport at Nanoscale Interfaces Laboratory Empa ‐ Swiss Federal Laboratories for Materials Science and Technology Dübendorf Switzerland; ^5^ Institute for Chemical and Bioengineering ETH Zürich Zürich Switzerland

**Keywords:** all‐perovskite tandems, doctor‐blade coating, flexible solar cells, mini‐modules, self‐assembled molecules, spatial uniformity, wide‐bandgap perovskites

## Abstract

Flexible all‐perovskite tandem solar modules offer high power‐to‐weight ratios, making them promising for applications in flexible electronics and aerospace. However, their fabrication over large area remains challenging due to difficulties in achieving uniform coating and controlled crystallization of perovskites using scalable meniscus‐based deposition methods, partly arising from poor ink‐substrate adhesion. Herein, perovskite ink wettability is improved by utilizing nickel oxide (NiO*_x_*) interlayer to suppress the aggregation of self‐assembled molecules (SAMs) and enhance the hydrophilicity of the SAM layer. As a result, wide‐bandgap (WBG) perovskite films were deposited onto large‐area flexible substrates via doctor‐blade coating, exhibiting excellent spatial uniformity and achieving minor pixel‐to‐pixel variation in power‐conversion efficiencies (PCEs) on cell scale. The best‐performing flexible WBG perovskite solar cells and mini‐modules achieve PCEs of 16.7% and 13.3%, respectively. Furthermore, when integrated with a blade‐coated narrow‐bandgap perovskite subcell, this strategy enables the fabrication of flexible all‐perovskite tandem solar cell with a PCE of 23.3%. An efficiency exceeding 20% is also demonstrated for flexible all‐perovskite tandem mini‐modules over an aperture area of 10.8 cm^2^. This study provides important insights into the regulation of SAM growth and establishes a foundation for the development of high‐efficiency flexible all‐perovskite tandem solar modules.

## Graphical abstract

Perovskite wettability on self‐assembled molecule (SAM) layers is improved by inhibiting SAM aggregation through the introduction of a NiO*_x_* interlayer. This enables spatially uniform coating of wide‐bandgap perovskite films onto 5 × 5 cm^2^ flexible substrate via doctor‐blade coating. Consequently, champion flexible wide‐bandgap perovskite and all‐perovskite tandem solar mini‐modules achieved efficiencies of 13.3% and 20.2%, respectively.

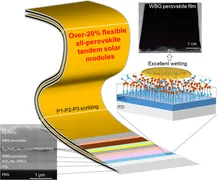

## 1 Introduction

Metal halide perovskite‐based photovoltaics have attracted widespread interest due to their high power‐conversion efficiencies (PCEs), potential for low‐cost fabrication, and broad applicability [1, 2, 3]. In recent years, perovskite‐based single‐junction and tandem devices have achieved significant advancements in efficiency, stability, and scalability [4, 5, 6, 7]. At the same time, many traditional photovoltaic enterprises and startups have been accelerating the commercialization of perovskite‐based photovoltaics. Compared with single‐junction perovskite solar cells (PSCs), constructing tandem solar cells (TSCs) by integrating a wide‐bandgap (WBG) perovskite top subcell with a narrow‐bandgap (NBG) bottom subcell can broaden absorption spectra and reduce thermalization loss, achieving much higher efficiencies [8]. When silicon or mixed Sn‐Pb perovskite is used as the absorber of the bottom subcell, corresponding TSCs have achieved the highest certified PCE of 35.2% and 30.1%, respectively, which are much higher than the 28% achieved by single‐junction PSCs [4].

Perovskites possess the significant advantage of solution processability over Si‐based photovoltaics, enabling the fabrication of lightweight, flexible solar cells at low cost with low energy consumption; making them compatible with high‐throughput roll‐to‐roll manufacturing [9, 10]. By combining the high efficiency of tandem devices with the lightweight nature of flexible devices, flexible all‐perovskite tandems can offer ultra‐high power‐to‐weight ratios compared with conventional photovoltaics and even perovskite/silicon tandem devices. These features make them particularly attractive for wearable electronics, portable electric chargers, building/vehicle‐integrated photovoltaics, and aerospace applications [11]. However, research on flexible all‐perovskite tandem devices remains rare, with the highest reported PCE of 28.2% achieved on small areas (<0.1 cm^2^), and 23% on mini‐module scale [12, 13]. Therefore, more effort is required on the scale‐up of flexible all‐perovskite tandems.

Although spin‐coated perovskite‐based single‐junction and tandem cells have achieved impressive progress, their performance typically deteriorates upon scaling to larger areas, particularly in tandem architectures [10, 14, 15]. The loss is primarily due to compromised uniformity of the perovskite absorber layer, charge‐transport layers, and charge‐selective interfaces, significantly hindering their commercialization prospects. Thus, developing scalable coating techniques capable of uniformly depositing perovskite films is critical. Industry‐compatible solution‐based methods, including spray coating, inkjet printing, doctor‐blade coating, and slot‐die coating, have been explored for depositing perovskite absorbers on large‐area substrates [10, 16]. In meniscus‐based coating processes like doctor‐blade and slot‐die coating, the wettability of perovskite inks on the substrates and synchronized crystallization mainly dominate the homogeneity of the resulting perovskite films [17]. To improve wetting behavior, strategies such as ink formulation engineering (e.g., tuning viscosity and surface tension) and substrate surface modification have been developed to enhance ink‐substrate adhesion [18].

Reducing ink surface tension is an effective approach to enhance the wettability, which has been achieved through the introduction of surfactants, such as l‐α‐phosphatidylcholine [19], and co‍‐‍solvents, such as 1,2‐dichlorobenzene [20]. Alternatively, modifying the substrate surface to be more hydrophilic can also improve perovskite wetting. Self‐assembled molecules (SAMs) have been widely used as hole‐selective contacts for efficient and stable PSCs [21]. To improve the wettability of perovskite inks on SAM layer, functionalisation of SAMs with hydrophilic groups and SAM blending has been widely proposed [22, 23, 24]. For instance, Zhang et al. utilized amphiphilic molecules as hole‐selective contacts, which displayed superwetting characteristics with regard to the perovskite precursor solution [25]. Magomedov et al. introduced 1,6 hexylenediphosphonic acid to (4‐(3,6‐dimethyl‐9H‐carbazol‐9‐yl)butyl)phosphonic acid (Me‐4PACz) precursor to enhance the hydrophilicity of SAM surface [22]. In addition, the substrates used for SAM anchoring also influences the wettability of perovskite ink on SAM layer [26, 27]. Improved wettability has been achieved by adding a metal oxide interlayer underneath the SAM to strengthen SAM anchoring. Tang et al. developed atomic layer deposited (ALD) indium tin oxide (ITO) to strengthen the SAM anchoring due to a higher density of anchoring sites, hydroxyl (─OH) groups, improving perovskite ink wettability [26]. Similarly, Yu et al. incorporated nickel oxide (NiO_*x*_) a buffer layer which enabled better wetting for perovskite growth, and the use of H_2_O_2_‐treated NiO_*x*_ nanoparticles further improved the wetting due to more ─OH groups on the surface [27]. Although substrate modifications have been successfully applied to improve the wetting of perovskite inks in previous works, the role of substrates (metal oxides) in regulating the growth of SAMs for favorable wetting of perovskite ink is not well understood. This is especially true for achieving spatial‐uniform perovskite films on large‐area flexible substrates using scalable coating methods.

In this work, we improved the perovskite wettability and coating uniformity via hole‐transport layer (HTL) modification. NiO_*x*_ interlayer was inserted between the flexible polyethylene naphthalate (PEN)/ITO substrate and SAM. We found that stronger interaction between NiO_*x*_ and SAM inhibits the aggregation of SAM molecules, facilitating the formation of more hydrophilic SAM surface. Therefore, the wettability of WBG perovskite ink on SAM is greatly improved, enabling uniform WBG perovskite films on 5 × 5 cm^2^ flexible substrates with great coverage and spatial homogeneity by doctor‐blade coating. The excellent spatial homogeneity of the perovskite film in terms of film thickness, morphology, crystallization and optoelectronic property was confirmed by various characterizations. As a result, flexible WBG PSCs achieved the highest PCE of 16.7% with minor pixel‐to‐pixel differences, and flexible WBG perovskite solar mini‐module achieved the highest efficiency of 13.3% over an aperture area of 10.8 cm^2^. When integrating WBG perovskite subcell and NBG perovskite subcell together, the best‐performing flexible all‐perovskite TSC achieved 23.3%. In addition, we also demonstrated a flexible all‐perovskite tandem solar mini‐module with a PCE of 20.2% over an aperture area of 10.8 cm^2^. This work brings new understanding of SAM growth regulation and highlights the crucial role of the NiO_*x*_/SAM bilayer in enabling uniform perovskite coating on large‐area flexible substrates, laying the foundation for achieving efficient, large‐area flexible all‐perovskite tandems.

## 2 Results and Discussion

### 2.1 Wetting Issue for Homogeneous Perovskite Coating by Doctor‐Blade Coating

**FIGURE 1:**
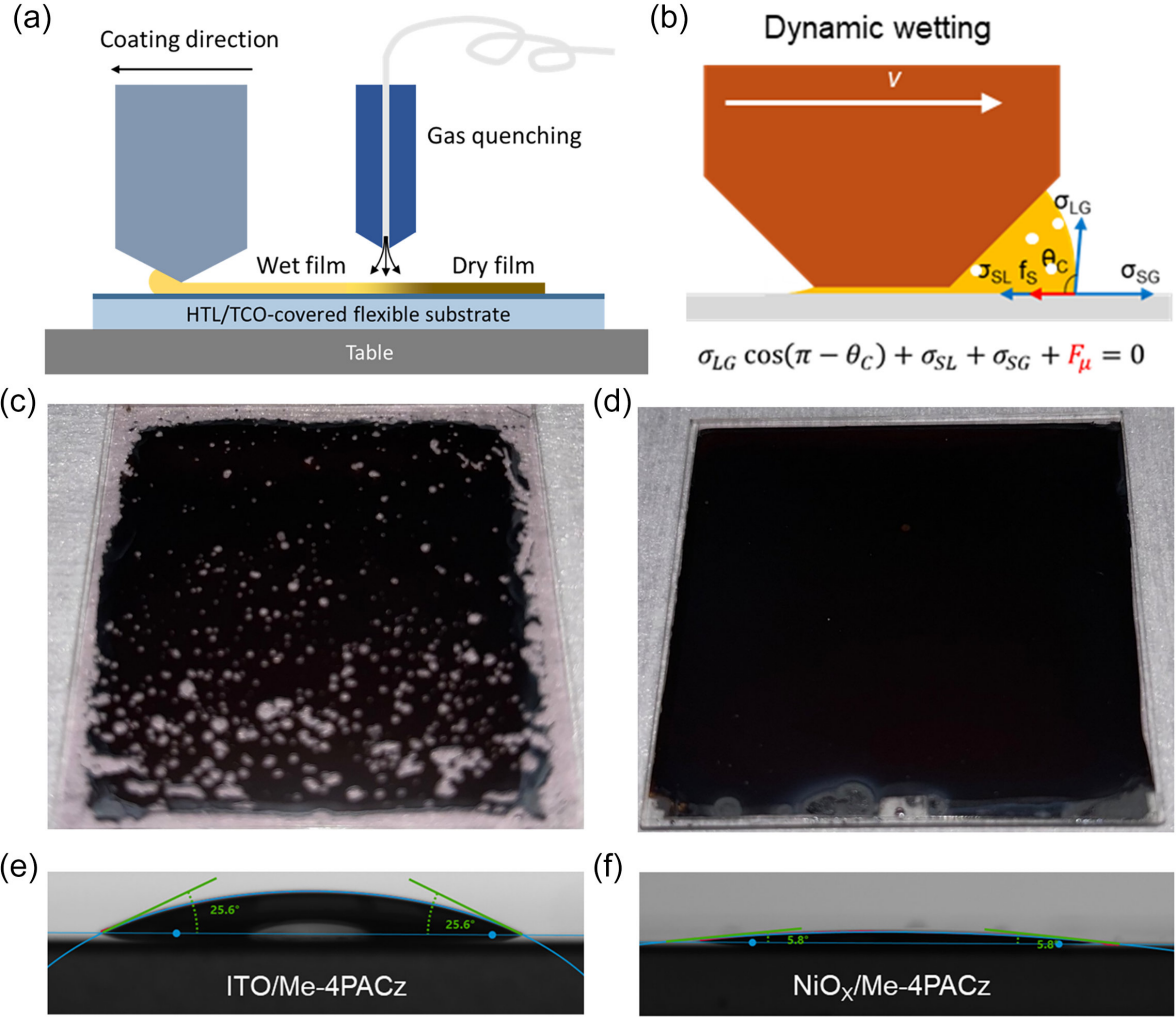
Wettability issue in doctor‐blade coating and its improvement. (a) Schematic diagram of perovskite deposition by doctor‐blade coating, followed by gas quenching. (b) Schematic diagram of dynamic wetting process during doctor‐blade coating, illustrating viscosity drag and air entrapment. (c,d) Optical images of blade‐coated WBG perovskite on 5 × 5 cm^2^ (c) ITO/Me‐4PACz and (d) ITO/NiO_*x*_/Me‐4PACz covered flexible substrates. (e,f) Representative contact angles of perovskite ink on Me‐4PACz‐covered (e) ITO and (f) ITO/NiO_*x*_.

To explore the potential of perovskite materials for flexible, lightweight applications, we aim to scale up the fabrication of flexible all‐perovskite tandems to make solar modules, starting from the uniform coating of WBG perovskite absorbers on flexible substrates. In this research, meniscus‐based doctor‐blade coating is used to deposit the perovskite absorber on the HTL‐covered flexible PEN/ITO substrates (Figure 1a). The wettability of the perovskite ink on substrates significantly influences the coating uniformity, crystallization, and thus device performance.

In the common static wetting regime, the contact angle is determined by the balance of intermolecular forces at the three‐phase contact line when the liquid droplet and the three‐phase contact line are at rest (Figure S1). In doctor‐blade coating, we must consider the dynamic wetting regime. Due to the relative motion between the blade and substrate, viscous forces arising from the internal friction of the ink introduce an additional resistance (viscous drag, *F*_µ_) to the flow. The flow dynamics is governed by the interplay between viscous stress and capillary forces, and the magnitude of viscous drag increases with coating speed [28, 29]. This viscous drag significantly affects dynamic wetting behavior and often leads to deteriorated wettability compared to static conditions (Figure 1b). Poor wettability of the perovskite ink on substrates in the dynamic wetting regime can induce air entrapment in the process of blade‐coating, resulting in void formation, non‐uniform film coverage, and increased interfacial defects [30, 31].

The poor wettability of the commonly used perovskite ink (perovskite precursors dissolved in mixed solvents of DMF and DMSO) on Me‐4PACz‐covered ITO substrates has been widely reported in spin‐coated perovskite films, causing de‐wetting and poor coverage (Figure S2) [22, 32]. On the other hand, during the blade‐coating process, the poor wettability leads to air entrapment and the formation of many voids within the perovskite film (Figure 1c,e). These challenges are even more pronounced on flexible polymeric substrates than on rigid glass, due to their surface unevenness, and weaker interaction between substrate and perovskite [12]. To improve the wettability of the perovskite ink, substrate modification was performed by introducing a NiO_*x*_ interlayer between the ITO and Me‐4PACz, significantly reducing the static contact angle from 27.2 ± 2.7° to 5.9 ± 1.3° (Figures 1e,f and S3). This enhanced wetting behavior facilitated the formation of well‐covered WBG perovskite films on flexible substrates by doctor‐blade coating (Figure 1d), meeting the prerequisites for the module fabrication.

### 2.2 Substrate Properties and Their Influence on SAM Growth

**FIGURE 2:**
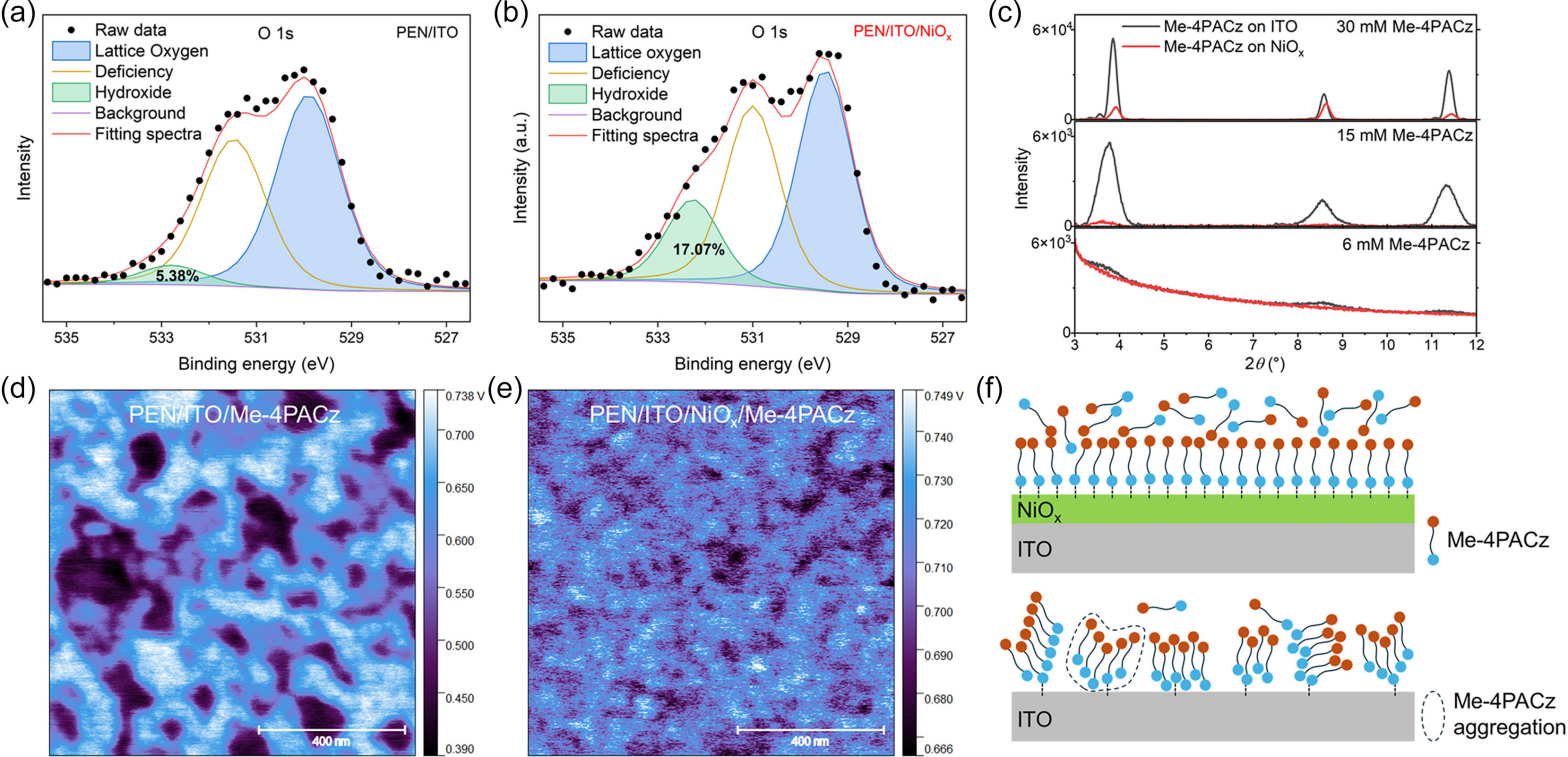
Substrate properties and their influence on SAM growth. (a,b) O 1s XPS core‐level spectra of (a) ITO and (b) ITO/NiO_*x*_ substrates. (c) XRD patterns of Me‐4PACz at different thickness coated on ITO and NiO_*x*_, with Me‐4PACz thickness controlled by concentration of Me‐4PACz solution (6, 15, 30 mM). (d,e) KPFM images of Me‐4PACz‐coated (d) ITO and (e) ITO/NiO_*x*_, showing the CPD distribution. (f) Schematic diagram of Me‐4PACz assembly on ITO and ITO/NiO_*x*_.

To understand the mechanism behind the wettability improvement, we first studied the basic properties of ITO and NiO_*x*_ films. Given that the hydroxyl (─OH) group plays a critical role in SAM anchoring, we conducted X‐ray photoelectron spectroscopy (XPS) on PEN/ITO substrate and PEN/ITO/NiO_*x*_ films, with the O 1s core‐level spectra shown in Figure 2a,b. The fitting results reveal three primary types of oxygen species corresponding to different chemical environments. The peaks in the low and high binding energy regions are mainly attributed to lattice oxygen and hydroxyl groups present on the surfaces of ITO and NiO_*x*_, respectively [26]. Compared with the bare ITO film, the NiO_*x*_ film exhibits a significantly higher proportion of hydroxyl‐derived oxygen species, increasing from 5.38% to 17.07%. In addition, due to the partial covalent character arising from d‐orbital participation and the smaller ionic radius of Ni, the bond strength between the SAM and NiO_*x*_ is stronger than that between SAM and ITO, which is also reported in literature [26, 33]. These differences in the amounts of anchoring sites and bonding strength significantly affects the growth of the Me‐4PACz molecules on substrates, further impacting the surface properties of the final Me‐4PACz films.

The crystallinity of the Me‐4PACz coated on ITO and NiO_*x*_ was investigated first via X‐ray diffraction (XRD) measurements (Figure 2c). Considering the difficulty in detecting ultra‐thin Me‐4PACz with only several molecular layers (often 1–3 nm) [34, 35], we intentionally enhanced the Me‐4PACz thickness by increasing the ink concentration for Me‐4PACz coating from 1 mM to 6, 15, and 30 mM. Interestingly, Me‐4PACz coated on ITO presents much higher peak intensities than that on NiO_*x*_ for all thicknesses measured, even on thin films (6 mM), indicating better crystallinity on ITO. The Me‐4PACz crystallization is mainly attributed to periodic π‐stacking among molecules [36]. Thus, we conclude that Me‐4PACz likely always exhibits more π‐stacking when coated on ITO than NiO_*x*_, even at the common thickness used in efficient PSCs (1 mM, ~1–3 nm). This was further supported by surface‐enhanced Raman scattering (SERS) measurements on thin (1 mM) Me‐4PACz films on ITO and ITO/NiO_*x*_ (Figure S4). Here, all vibrational modes exhibited a reduction in intensity upon the introduction of NiO_*x*_ interlayer, suggesting a decrease in the crystallinity of the Me‐4PACz film.

In order to investigate the influence of the NiO_*x*_ interlayer on the surface potential and spatial uniformity of the Me‐4PACz layer, Kelvin probe force microscopy (KPFM) measurements were performed in an N_2_‐filled glovebox. Contact potential difference (CPD) maps are acquired for bare ITO, bare ITO/NiO_*x*_, Me‐4PACz‐coated ITO, and Me‐4PACz‐coated ITO/NiO_*x*_ surfaces. Absolute work function (WF) values were derived from the measured CPD using a tip work function of 4.5 eV, which was calibrated against freshly cleaved highly oriented pyrolytic graphite prior to each measurement session.

The CPD ranges of the bare ITO and ITO/NiO_*x*_ surfaces are comparable, at approximately 73 and 83 mV, respectively. The distributions are narrow and symmetric, reflecting the intrinsic granular structure of each substrate. Correspondingly, the average WF values are 4.87 eV for ITO and 4.83 eV for ITO/NiO_*x*_, in good agreement with literature reports [37, 38]. However, when Me‐4PACz is deposited on ITO, the CPD range increases substantially to ~350 mV. Distinct low‐CPD regions are clearly visible in Figures 2d and S5, indicative of molecular aggregation and incomplete surface coverage. This is further supported by increased surface roughness after Me‐4PACz coating on ITO (Figure S6). By contrast, depositing Me‐4PACz on NiO_*x*_ yields a CPD range of only ~83 mV, comparable to that of the bare ITO/NiO_*x*_ surface (Figures 2e and S5). This confirms that the NiO_*x*_ interlayer effectively suppresses Me‐4PACz aggregation and promotes uniform coverage. Notably, the mean WF shift upon Me‐4PACz deposition on NiO_*x*_ is +378 mV, uniformly raising the WF from 4.83 to 5.21 eV across the entire surface. On ITO, the mean WF shift is not representative due to the large lateral CPD inhomogeneity. The high‐CPD regions correspond to areas of intact Me‐4PACz coverage and reach a local WF of ~5.24 eV, which is comparable to that achieved uniformly on NiO_*x*_. However, the low‐CPD areas pull the surface average down significantly. This demonstrates that the intrinsic WF of Me‐4PACz is similar on both substrates, but only the NiO_*x*_ interlayer enables this WF to be established homogeneously across the full substrate area. Such improved homogeneity is expected to suppress interfacial recombination, enhance hole extraction, and achieve better device stability [39, 40].

With these results, we hypothesize that the competition between substrate/Me‐4PACz interaction and Me‐4PACz self‐interaction is the dominant factor influencing the growth of Me‐4PACz molecules, as previously suggested by theoretical modeling studies [41, 42]. On NiO_*x*_, the self‐interaction among Me‐4PACz molecules is suppressed due to stronger bonding and more anchoring sites, resulting in poorer crystallinity and more pronounced amorphous characteristics. By contrast, ITO provides weaker bonding and fewer anchoring sites, allowing Me‐4PACz self‐interactions to dominate and promoting molecular aggregation for crystallization, proved by XRD results (Figure 2c). The less‐aggregated Me‐4PACz on NiO_*x*_ therefore has greater spatial uniformity than that Me‐4PACz on ITO, proved by KPFM results (Figure 2d,e). Considering this, and the multilayer structure of SAMs, schematic diagrams of Me‐4PACz assembly on ITO and NiO_*x*_ are shown in Figure 2f. Less‐aggregated and amorphous Me‐4PACz layer increases its chance to expose more hydrophilic groups (e.g., ─H_2_PO_3_) toward perovskite, beneficial for improved wettability. To gain even deeper insight into the underlying mechanism, some surface‐sensitive characterization techniques, such as angle‐resolved XPS and Near‐edge X‐ray absorption fine structure spectroscopy, could be useful to effectively establish the direct relationship between surface composition, molecular orientation and surface hydrophilicity [43, 44].

Overall, inserting NiO_*x*_ interlayer suppresses the aggregation of Me‐4PACz molecules and promotes a spatially uniform distribution of Me‐4PACz on substrates, thereby enabling better wettability for perovskite inks. Although our previous discussion highlighted the improved wettability of perovskite ink on Me‐4PACz by inserting NiO_*x*_ interlayer, we also found that this approach is generalizable to other SAMs. A similar study on (4‐(7H‐dibenzo[c,g]carbazol‐7‐yl)butyl)phosphonic acid (4PADCB) demonstrated that the NiO_*x*_ interlayer likewise enhances perovskite wetting (Note S1). This improvement facilitates uniform coating of perovskite absorbers on several NiO_*x*_/SAM substrates, including NiO_*x*_/4PADCB, NiO_*x*_/2PACz, and NiO_*x*_/MeO‐2PACz with good coverage. Four different SAMs, Me‐4PACz, 4PADCB, 2PACz, and MeO‐2PACz, were screened by evaluating their effect on charge‐carrier dynamics at hole selective interfaces. Based on time‐resolved photoluminescence (TRPL) and photoluminescence quantum yield (PLQY) results, Me‐4PACz was chosen as SAM to pair with NiO_*x*_ as HTL for subsequent fabrication of perovskite films and devices (Note S2).

### 2.3 Homogeneity Evaluation of Flexible WBG Perovskite Films

As discussed above, the introduction of the NiO_*x*_ interlayer modulates the SAM growth, increases the surface hydrophilicity, and significantly improves the wettability of the perovskite ink, thereby enabling the deposition of flexible WBG perovskite films with excellent coverage. Furthermore, among the NiO_*x*_/SAM bilayers examined in this study, NiO_*x*_/Me‐4PACz‐based perovskite films also exhibited superior optoelectronic properties (Note S2). To further evaluate the changes in film uniformity after substrate modification, spatially resolved characterization techniques were employed. The relative standard deviation (RSD) was used as a quantitative metric to assess the spatial homogeneity of the flexible WBG perovskite films.

**FIGURE 3:**
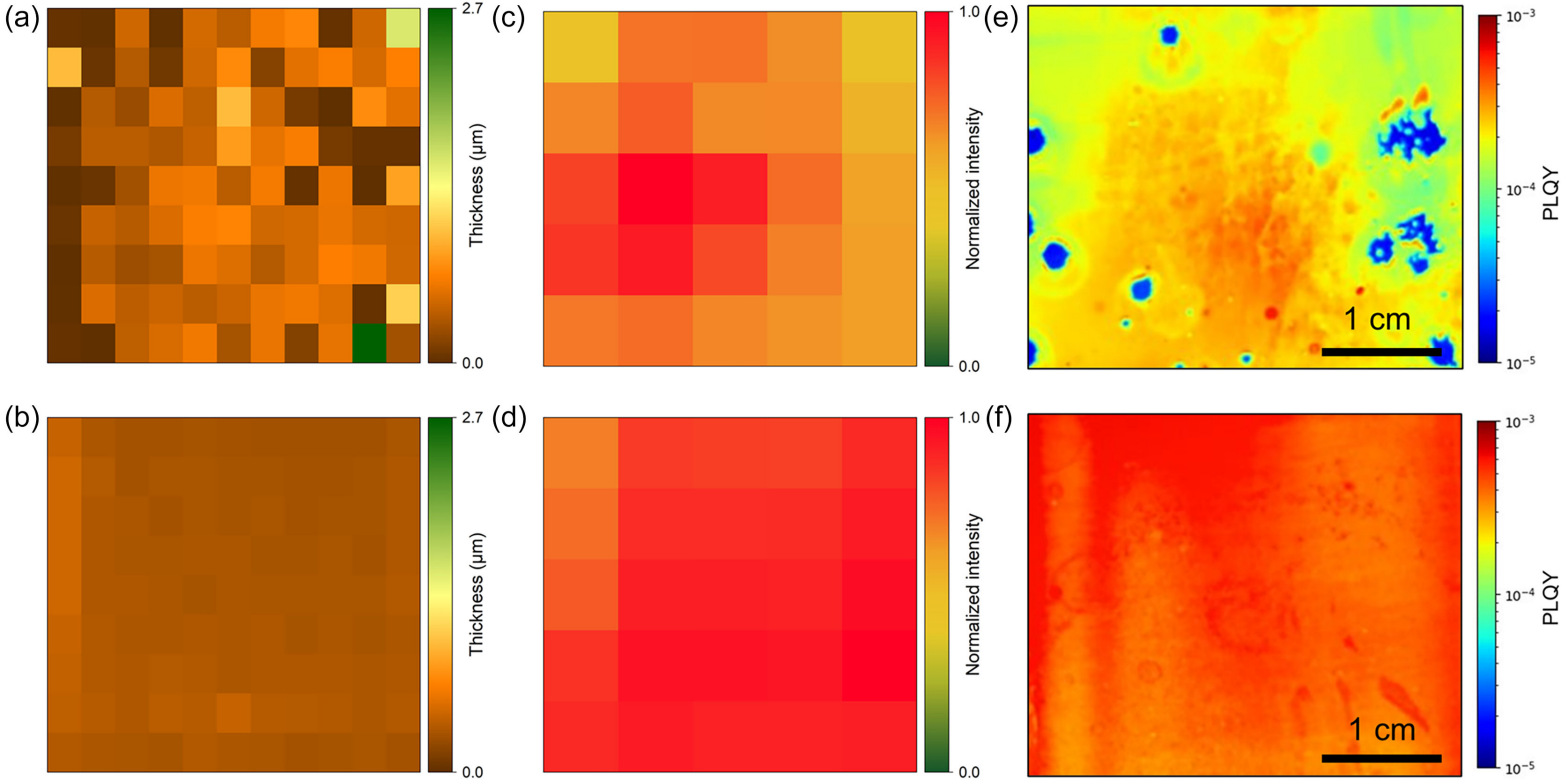
Homogeneity evaluation of flexible WBG perovskite films. Heatmaps of 5 × 5 cm^2^ flexible WBG perovskite films: (a,b) thickness derived from XRF measurements on (a) reference and (b) NiO_*x*_/Me‐4PACz‐based films. (c,d) Normalized intensity of the perovskite (100) peak obtained from XRD measurements on (c) reference and (d) NiO_*x*_/Me‐4PACz‐based films. (e,f) PLQY images of (e) reference and (f) NiO_*x*_/Me‐4PACz‐based films, recorded over 3.6 × 3 cm^2^ regions at the center.

Achieving high spatial uniformity in thickness is a critical prerequisite for obtaining homogeneous optoelectronic properties in perovskite films. X‐ray fluorescence (XRF) mapping was conducted on 5 × 5 cm^2^ flexible WBG perovskite films at 9 × 11 spatial positions to determine the elemental spatial distribution and thickness of films. ITO/Me‐4PACz‐based perovskite film exhibited pronounced nonuniformity in Pb content, which was significantly mitigated by introducing a NiO_*x*_ interlayer (Figure S11). Furthermore, thickness heatmaps derived from XRF results reveal a substantial improvement in thickness uniformity, with the RSD decreasing markedly from 83.87% to 11.84% (Figure 3a,b).

Furthermore, spatial uniformity of the perovskite films in terms of crystallinity was measured by XRD mapping on both reference and NiO_*x*_/Me‐4PACz‐based perovskite film. All XRD patterns are presented in Figure S12, collected from 5 × 5 spatial positions across each film (Figure S13a). The peak area and full width at half maximum (FWHM) of the perovskite (100) diffraction peak were extracted by fitting each XRD pattern with Gaussian functions. All peak area data were normalized according to the maximum value. In terms of the normalized peak area of the perovskite (100) diffraction peak, the NiO_*x*_/Me‐4PACz‐based perovskite film exhibited significantly improved homogeneity compared with the reference film, with the RSD decreasing from 12.9% to 6.8% (Figure 3c,d). Moreover, the modified film also showed better FWHM uniformity, with a lower RSD of 3.1% compared with 4.3% for the reference (Figure S13b,c). These results collectively confirm that the NiO_*x*_/Me‐4PACz‐based perovskite film possesses better homogeneity in crystallinity.

Scanning electron microscopy (SEM) images were captured to assess the morphological homogeneity of the NiO_*x*_/Me‐4PACz‐based perovskite film. These images were acquired from four distinct locations across the 5 × 5 cm^2^ perovskite films (Figure S14), and all images revealed consistent surface morphology, characterized by dark perovskite grains and bright PbX_2_ (X = I, Br) particles localized at the grain boundaries [45]. The grain sizes were statistically analyzed and fitted with a normal distribution, showing comparable grain size distributions across the different regions, with an average grain size of approximately 190 nm and a standard deviation of ~ 68 nm. This consistency in morphology indicates homogeneous crystallization behavior, aligning well with XRD mapping results (Figures 3d and S13), which is attributed to enhanced wettability, alongside proper coating, and drying parameters for deposition of WBG perovskite films.

Finally, PLQY imaging was performed to assess the spatial uniformity of the optoelectronic property of flexible WBG perovskite films (Figure 3e,f). The NiO_*x*_/Me‐4PACz‐based WBG perovskite film exhibits significantly improved homogeneity compared to the reference film, which aligns well with XRF and XRD mapping results. Moreover, PLQY values are also improved after introducing NiO_*x*_ interlayer. We attribute this to improved Me‐4PACz coverage on substrates, which inhibits interfacial recombination at the buried interface. These improvements in both PLQY and its spatial uniformity are critical for the development of efficient solar modules.

### 2.4 PV Performance of Single‐Junction WBG PSCs and Mini‐Modules

**FIGURE 4:**
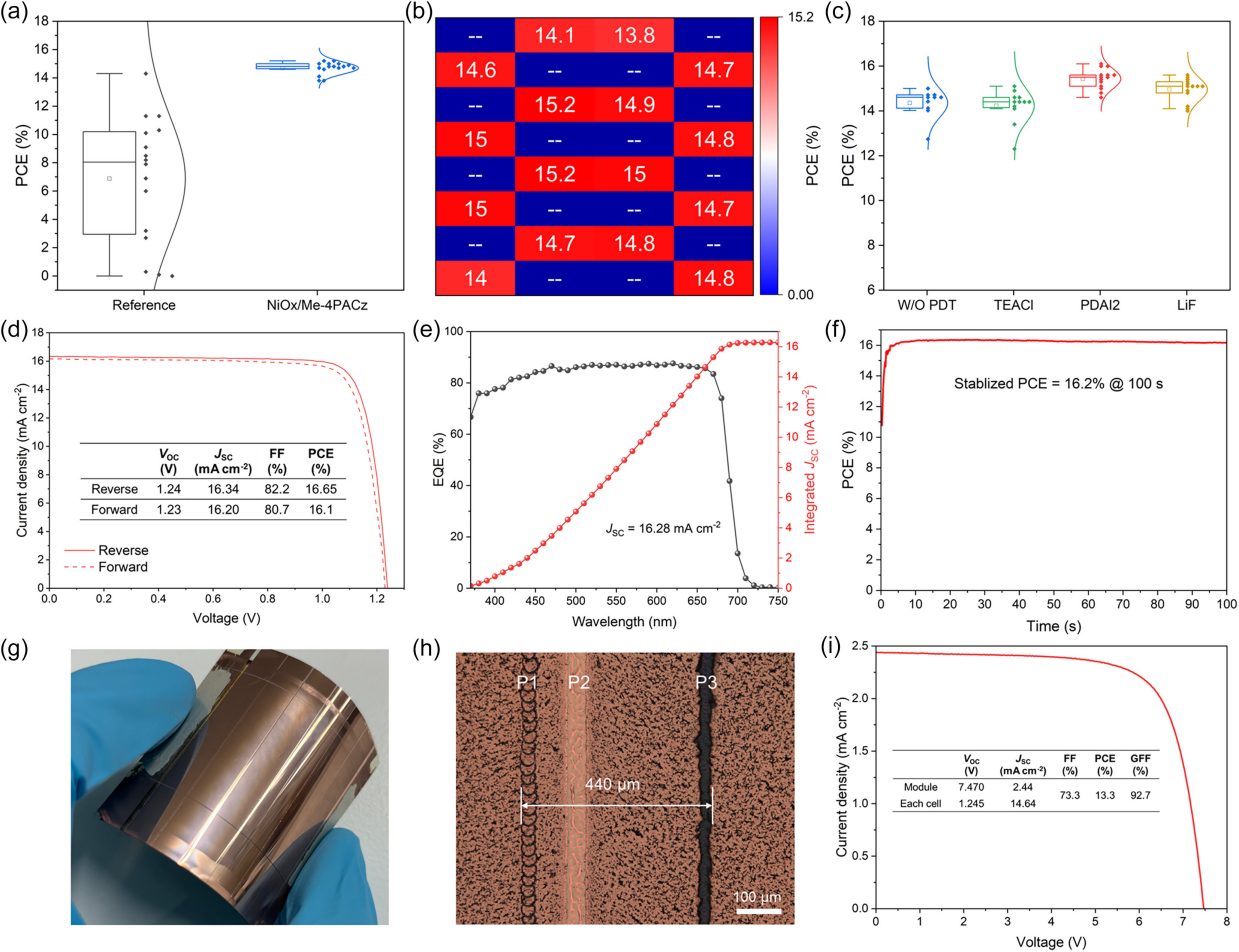
PV performance of flexible WBG PSCs and mini‐modules. (a) Statistical PCE of reference and NiO_*x*_/Me‐4PACz flexible WBG PSCs. Data points were taken from two samples,16 pixels for each. (b) PCE heatmap of representative NiO_*x*_/Me‐4PACz flexible WBG PSCs fabricated on one 5 × 5 cm^2^ PEN/ITO substrate. (c) Statistical PCE of typical flexible WBG PSCs with different PDT strategies. (d) *J–V* curves, (e) EQE spectra, and (f) Steady‐state efficiency output of the best‐performing flexible WBG PSC. (g) Photograph of a 5 × 5 cm^2^ flexible WBG perovskite solar mini‐module. (h) Micrograph of P1‐P2‐P3 scribe lines used to determine GFF of mini‐module. (i) *J*‐*V* curve of the best‐performing flexible WBG perovskite solar mini‐module.

Flexible WBG PSCs were fabricated first with the configuration of PEN/ITO (or ITO/NiO_*x*_)/Me‐4PACz/perovskite/C_60_/bathocuproine (BCP)/Cu. To assess spatial homogeneity in device performance, 16 pixels were patterned across the 5 × 5 cm^2^ flexible substrates, as illustrated in Figure S15. The statistical photovoltaic (PV) parameters, including PCE, open‐circuit voltage (*V*_OC_), short‐circuit current density (*J*_SC_), and fill factor (FF), for both reference and NiO_*x*_/Me‐4PACz‐based devices are shown in Figures 4a and S16. Compared with the reference sample, the introduction of the NiO_*x*_ interlayer significantly reduced the variation in device performance across the samples. A PCE heatmap across the NiO_*x*_/Me‐4PACz‐based sample (Figure 4b) demonstrates the excellent spatial uniformity, with minimal pixel‐to‐pixel variation, as evidenced by a low RSD of 2.86%. The best‐performing pixel from the NiO_*x*_/Me‐4PACz‐based sample exhibited a greater PCE of 15.2% surpassing 14.3% for the reference sample.

To further enhance the performance of flexible WBG PSCs, postdeposition treatments (PDT) were explored for perovskite surface passivation. Three materials, tetraethylammonium chloride (TEACl), 1,3‐propane diammonium iodide (PDAI_2_), and lithium fluoride (LiF), were screened for post‐treatment via blade‐coating (TEACl and PDAI_2_) or thermal evaporation (LiF) prior to C_60_ deposition. The statistical distribution of PCEs for devices treated with each material is presented in Figure 4c. Among them, the PDAI_2_‐treated flexible WBG PSCs demonstrated the highest average PCE, primarily due to improvements in *V*_OC_ and FF (Figure S17). Note that PDAI_2_‐treatment improves the PLQY without the sacrifice of film uniformity (Figure S18). This enhancement is attributed to suppression of nonradiative recombination and facilitation of efficient charge transfer from the perovskite layer to C_60_ [46]. The current density–voltage (*J–V*) characteristics of the best‐performing flexible WBG PSC, measured under simulated AM 1.5G solar illumination (100 mW cm^−2^), are shown in Figure 4d. The best‐performing cell achieved a PCE of 16.7% at reverse (16.1% at forward) voltage scan, with a *J*_SC_ of 16.34 (16.20) mA cm^−2^, a *V*_OC_ of 1.24 (1.23) V, and an FF of 82.2% (80.7%). The external quantum efficiency (EQE) spectrum yielded an integrated *J*_SC_ over the entire solar spectrum of 16.28 mA cm^−2^ (Figure 4e), in agreement with the *J*_SC_ obtained from the *J–V* measurements. The steady‐state efficiency output was achieved by tracking at the maximum power point for 100 s, which gives an efficiency of 16.2%, comparable to PCE from the *J–V* measurements (Figure 4f).

The mechanical durability of flexible WBG PSCs were monitored after bending under compressive and tensile stress. The flexible WBG PSC can maintain 89% of its initial performance after 2000 bending cycles under compressive stress with a bending radius of 9 mm (Figure S19). By contrast, the device is much less stable under tensile stress at the same bending radius, with only 25% of the initial performance kept after 400 bending cycles. This bending radius corresponds to a strain of 0.7%, according to the formula: *ɛ* = *t*/2*R*, where *ɛ* is the strain, *t* is the thickness of PEN/ITO foil (125 µm), and *R* is the bending radius. The pronounced efficiency loss of flexible WBG PSC under tensile stress is mainly attributed to crack formation during cyclic bending, which increases the series resistance, whereas the surface morphology remains essentially intact after designed cyclic bending test under compressive stress (Figure S20). We also evaluated the operational stability of flexible WBG PSCs under maximum‐power‐point (MPP) tracking. The encapsulated device can maintain 80% of its initial performance after 168 h MPP tracking at 65 °C in ambient air (ISOS‐L‐2 protocol) (Figure S21).

Encouraged by the notable improvements in film uniformity and device efficiency, we extended our efforts toward the fabrication of flexible WBG perovskite solar mini‐modules The conventional P1‐P2‐P3 laser scribing process was employed, with 6 mm width × 3 cm length for an individual cell (Figure S22). Each mini‐module comprises six series‐connected cells and have a total aperture area of 10.8 cm^2^ (Figure 4g). The interconnection gap (dead area) between cells was limited to 440 µm, corresponding to a geometric fill factor (GFF) of 92.7% (Figure 4h). With this approach, multiple batches of flexible WBG perovskite solar mini‐modules were fabricated, yielding an average PCE of 11.6% (Figure S23). The best‐performing mini‐module achieved a PCE of 13.3%, with a *J*_SC_ of 2.44 mA cm^−2^, a *V*_OC_ of 7.47 V, and an FF of 73.3% (Figure 4i), with a steady‐state efficiency of 12.9% (Figure S24). The average *J*_SC_ and *V*_OC_ of these series‐connected cells were calculated to be 14.64 mA cm^−2^ (15.79 mA cm^−2^ in active area) and 1.25 V, respectively, which are almost consistent with those values achieved in small‐area cells. The observed cell‐to‐module loss in PCE is primarily attributed to the reduction of FF. The loss can partially originate from increased series resistance associated with the bottom ITO electrode and the ITO/Cu interconnections formed through the P2 trench. Planar light‐emitting image of a flexible WBG perovskite mini‐module also exhibits good spatial homogeneity (Figure S25), aligning well with the excellent spatial uniformity of the flexible perovskite films shown by different mapping techniques and the minor spatial variation in cell performance.

### 2.5 PV Performance of Flexible All‐Perovskite Tandems

**FIGURE 5:**
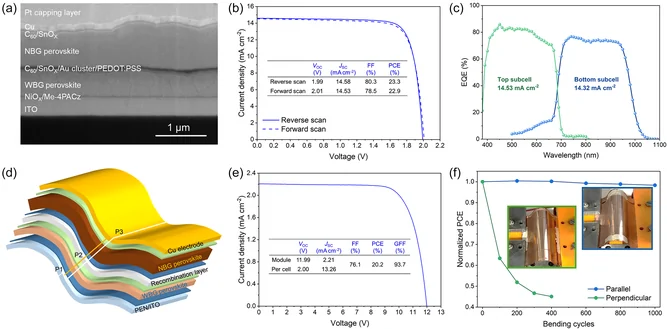
Performance of flexible all‐perovskite tandems. (a) Cross‐sectional FIB‐SEM image of flexible all‐perovskite TSC. (b) *J–V* curves and (c) EQE spectra of the best‐performing flexible all‐perovskite TSC, including the integrated *J*_SC_s of both top and bottom subcells. (d) Schematic diagram of flexible all‐perovskite tandem solar module. (e) *J–V* curve of the best‐performing flexible all‐perovskite tandem solar mini‐module. (f) Mechanical bending durability of unencapsulated flexible all‐perovskite tandem solar mini‐modules under tensile stress, with the bending direction oriented perpendicular or parallel to the scribe lines. The inset shows the photographs of mini‐modules in curved state under different bending scenarios.

After successful fabrication of flexible WBG perovskite solar mini‐modules with high efficiency, we further demonstrated flexible all‐perovskite tandem solar mini‐modules by integrating WBG perovskite subcell with a blade‐coated FA_0.7_MA_0.3_Sn_0.5_Pb_0.5_I_3_ NBG (~1.26 eV) perovskite subcell in series. The single‐junction flexible NBG PSCs used to construct the tandem devices exhibited a PCE of 17.8%, along with a *V*_OC_ of 0.80 V, a *J*_SC_ of 28.60 mA cm^−2^, and a FF of 77.3% (Figure S26a). Its corresponding EQE‐integrated *J*_SC_ is 28.75 mA cm^−2^ (Figure S26b). Figure 5a shows the cross‐sectional SEM image of a flexible tandem cell with a configuration of PEN/ITO/NiO_*x*_/Me‐4PACz/WBG perovskite/C_60_/SnO_*x*_/Au cluster (1.2 nm)/PEDOT:PSS/NBG perovskite/C_60_/SnO_*x*_/Cu.

First, the performance of flexible all‐perovskite TSCs was evaluated; 50 flexible all‐perovskite TSCs from multiple batches delivered an average efficiency of 21.1% (Figure S27). The *J–V* characteristic of the best‐performing cell shows that it achieved a PCE of 23.3% under reverse (22.9% under forward) voltage scan, with a *J*_SC_ of 14.58 (14.53) mA cm^−2^, a *V*_OC_ of 1.99 (2.01) V, and an FF of 80.3% (78.5%) (Figure 5b). From EQE measurements, the integrated *J*_SC_s are 14.53 mA cm^−2^ for the WBG perovskite top subcell and 14.32 mA cm^−2^ for the NBG perovskite bottom subcell (Figure 5c), in close agreement with the *J*_SC_ values obtained from *J–V* measurement. The steady‐state efficiency output was achieved by tracking at the maximum power point for 100 s, which gives an efficiency of 22.3% (Figure S28). Further performance improvement is constrained by significant *V*_OC_ losses in both subcells, as well as a limited *J*_SC_ contribution from the bottom subcell, mainly due to insufficient suppression of nonradiative recombination in both WBG and NBG perovskite absorbers. More research works on regulating crystallization and passivating defects of WBG and NBG perovskites, via meniscus‐based coating method, are required to further boost the performance of flexible all‐perovskite TSCs. According to ISOS‐L‐2 protocol, encapsulated flexible all‐perovskite TSC can maintain 75.2% of peak PCE after 51 h MPP tracking (Figure S29).

Flexible all‐perovskite tandem mini‐modules were also fabricated via a conventional P1‐P2‐P3 laser scribing process developed for flexible WBG mini‐modules, with its schematic diagram shown in Figure 5d. The interconnection (dead) area exhibits a width of 380 µm, corresponding to a geometric fill factor (GFF) of 93.7% (Figure S30). As a result, a flexible all‐perovskite tandem solar mini‐module was demonstrated with a high PCE of 20.2%, an excellent FF of 76.1% (Figure 5e), and a stabilized PCE of 19.2% (Figure S31).

We further evaluated mechanical durability of flexible all‐perovskite tandem solar mini‐modules under tensile stress. When bent parallel to the scribe lines (minimum bending radius of 9 mm), the device retained over 98% of their initial performance after 1000 bending cycles (Figure 5f). In contrast, bending perpendicular to the scribe lines resulted in a pronounced degradation, with only 45% of the initial performance remaining after 400 cycles. This substantial reduction in PCE is attributed to formation of cracks during cyclic deformation (Figure S32a). The cracks, oriented perpendicular to the current flow, increase the series resistance and thus impair device performance (Figure S33). Under milder bending condition (perpendicular to the scribe lines, minimum bending radius of ~15 mm, and strain of ~0.4%), the mini module can still maintain 97% of their initial performance after 3000 cycles (Figure S34). The significantly improved bending durability compared with that at a bending radius of 9 mm is attributed to the avoidance of crack formation under reduced mechanical strain (Figure S32b).

To further improve the mechanical durability of flexible all‐perovskite tandems to meet the requirements for future practical applications, the following three strategies are particularly promising: (1) replacing brittle ITO with alternative transparent electrodes, such as silver nanowires, graphene, or conductive polymers [47]; (2) reducing residual strain within the perovskite absorber and introducing flexible molecular “binders” at perovskite grain boundaries and charge‐selective interfaces to enhance strain tolerance and suppress interlayer delamination [48, 49, 50]; and (3) positioning the perovskite absorbers and other functional layers close to the neutral mechanical plane through rational design of the device substrate and encapsulation [51]. Furthermore, depending on the intended practical application, careful attention should be paid to the design and layout of flexible modules to minimize exposure to adverse bending conditions.

## 3 Conclusion

In summary, we elucidated the critical role of the substrate in regulating the growth of SAM molecules, governed by the competition between SAM self‐interaction and substrate‐SAM interaction. By introducing a NiO_*x*_ interlayer, the hydrophilicity of the Me‐4PACz layer was enhanced through suppression of molecular aggregation, thereby enabling the deposition of spatially uniform WBG perovskite films on large‐area flexible substrates via doctor‐blade coating. As a result, the flexible WBG perovskite films exhibited significantly improved uniformity in thickness, crystallinity, and optoelectronic property. Consequently, flexible WBG (~1.8 eV) PSCs and corresponding mini‐modules achieved the highest PCEs of 16.7% and 13.3%, respectively, which are among the highest reported efficiencies (Figure S35). By integrating the optimized WBG perovskite top subcell with a blade‐coated NBG perovskite bottom subcell, all‐perovskite TSCs reached a peak PCE of 23.3%. Furthermore, we demonstrated a flexible all‐perovskite tandem solar mini‐module with a PCE of 20.2% over an aperture area of 10.8 cm^2^. This study provides important insights into the regulation of SAM growth and establishes a foundation for the development of high‐efficiency flexible all‐perovskite tandem solar modules.

## Funding

This work was supported by the Horizon Europe Research and Innovation Program (101075605, 101147653); Schweizerischer Nationalfonds zur Förderung der Wissenschaftlichen Forschung (200021_213073, 200021L_219983, 200021_231373); Bundesamt für Energie (SI/502549‐01); Eurostars; Werner Siemens‐Stiftung.

## Conflicts of Interest

The authors declare no conflicts of interest.

## Supporting information

Supplementary Material

## Data Availability

The data that support the findings of this study are available from the corresponding author upon reasonable request.
