# Supplementary material for: Scalable Coating of Spatially Uniform WBG Perovskites via Inhibiting SAM Aggregation for Flexible All‐Perovskite Tandem Modules
Source: Small Sci. 2026 Aug 14;6(8):e70379. doi: 10.1002/smsc.70379 (PMC13489340; doi:10.1002/smsc.70379)
Supplement: Supplementary file 1 [file SMSC-6-e70379-s001.pdf]

Scalable Coating of Spatially Uniform WBG Perovskites via Inhibiting SAM Aggregation for Flexible All-Perovskite Tandem Modules

Jincheng Luo[1], Jonathan S. Austin[1,*], Alessia Romio[1], Antonio Cabas Vidani[2], Huagui Lai[1], Cédric Bühler[3], Stefano Saroglia[1], Pedro Octavio Quintana Ceres[1], Mirjana Dimitrievska[4], Thilo Glatzel[3], Chih-Jen Shih[5], Fan Fu[1,*]

[1]Laboratory for Thin Films and Photovoltaics, Empa - Swiss Federal Laboratories for Materials Science and Technology, Dübendorf, 8600, Switzerland

[2]Fluxim AG, Winterthur, 8400, Switzerland

[3]Department of Physics, University of Basel, Basel, 4056, Switzerland

[4]Transport at Nanoscale Interfaces Laboratory, Empa - Swiss Federal Laboratories for Materials Science and Technology, Dübendorf, 8600, Switzerland

[5]Institute for Chemical and Bioengineering, ETH Zürich, Zürich, 8093, Switzerland

[*]Corresponding author: jonathan.austin@empa.ch; fan.fu@empa.ch

Table-of-Contents

Materials and methods	Page 3-8
Figure S1-6	Page 9-14
Note S1-2, Figure S7-10	Page 15-20
Figure S11-35	Page 21-45
Note S3	Page 46
Table S1-2	Page 47-48
References	Page 49

Materials and methods

Materials

All materials were used as received without further purification. N,N-Dimethylformamide (DMF, 99.8 %), dimethyl sulfoxide (DMSO, 99.5 %), chlorobenzene (CB, 99.8%), isopropanol (IPA, 99.5 %), propane-1,3-diammonium iodide ($PDAI_2$, 98%), tin iodide (SnI_2, 99.99%), tin fluoride (SnF_2, 99%), tin powder (99.5%), Lithium fluoride (LiF, 99.99%), and lead thiocyanate ($Pb(SCN)_2$) were purchased from Sigma-Aldrich Pty Ltd. PbI_2 (99.5 %), $PbBr_2$ (99.999 %), CsI (99.999 %), (4-(3,6-Dimethyl-9H-carbazol-9-yl)butyl)phosphonic acid (Me-4PACz), (2-(9H-carbazol-9-yl)ethyl)phosphonic acid (2PACz), and (2-(3,6-Dimethoxy-9H-carbazol-9-yl)ethyl)phosphonic acid (MeO-2PACz), were purchased from Tokyo Chemical Industry Co., Ltd.. (4-(7H-dibenzo[c,g]carbazol-7-yl)butyl)phosphonic acid (4PADCB) was purchased from Luminescence Technology Corp. Methylammonium iodide (MAI, >99.99 %), Formamidinium iodide (FAI, >99.99 %), and 2-thiopheneethylammonium chloride (TEACl) were purchased from Greatcell Solar Materials. Fullerene (C_{60}) and bathocuproine (BCP) were purchased from Xi'an Yuri Solar Co. Ltd. PEDOT:PSS (Clevios PVP Al 4083) was purchased from Heraeus Epurio LLC. Ethanol (anhydrous, ≥99.9%) was purchased from VWR International, LLC. PEN/ITO substrates and NiO_x nanoparticles were purchased from Liaoning Youxuan New Energy Technology Co., Ltd.

Solution preparation

The WBG $FA_{0.8}Cs_{0.2}Pb(I_{0.6}Br_{0.4})_3$ (1.2 M) perovskite precursor was prepared by dissolving 264.24 mg $PbBr_2$, 221.28 mg PbI_2, 165.12 mg FAI, 62.35 mg CsI, and 1.94 mg $Pb(SCN)_2$ in 1 mL mixed solution of DMF and DMSO (19:1, v/v). The perovskite precursor was stirred at 45 °C for 2 h and filtered through a 0.22 μm PTFE filter before use. NiO_x dispersion was prepared by dissolving NiO_x nanoparticle in deionized water with a concentration of 12 mg/mL. Me-4PACz solution was prepared by Me-4PACz in ethanol with a concentration of 0.4 mg/mL. $PDAI_2$ solution was prepared by dissolving 1 mg $PDAI_2$ in 1 mL IPA.

The NBG Sn-Pb $FA_{0.7}MA_{0.3}Pb_{0.5}Sn_{0.5}I_3$ (1.8 M) perovskite precursor was prepared by re dissolving 414.91 mg of PbI_2, 335.27 mg of SnI_2, 216.68 mg of FAI, 85.84 mg of MAI, 7.05 mg of SnF_2 and 3 mg of tin powder in 1 mL mixed solution of DMF and DMSO (DMF: DMSO = 22: 3, v/v). Additive D-Homoserine Lactone hydrochloride (1 mol%) was added in the precursor solution. $EDAI_2$ solution was prepared by dissolving 1 mg $EDAI_2$ in 1 mL IPA and 1 mL CB.

Fabrication for WBG PSCs

Prepatterned PEN/ITO substrates were first fixed onto rigid glass plates with glue and then cleaned in IPA, water with detergent, water, and IPA sequentially by sonication for 15 min. Before device fabrication, the substrates were further dried by N_2 flow and cleaned by UV/Ozone treatment (Jelight Company Inc.) for 30 min. A water-based NiO_x dispersion (12

mg/mL) was spin-coated onto the cleaned PEN/ITO substrates at 3000 rpm for 30 s, followed by annealing at 110 °C for 15 minutes. The scalable deposition of NiO_x is discussed in **Note S3**. Then, PEN/ITO/NiO_x stacks were immediately transferred to the glovebox for SAM deposition. 80 μL of SAM solution, such as 2-(9H-carbazol-9-yl)ethyl]phosphonic acid (Me-4PACz), was blade-coated onto the PEN/ITO/NiO_x with a gap of 250 μm at a coating speed of 6 mm/s, followed by gas quenching by N_2 stream under a pressure of 5 psi. Samples were then transferred to a preheated hotplate at 100 °C for 10 minutes of annealing. After cooling down, 60 μL of WBG perovskite ink was blade-coated with a coating gap of 350 μm at a coating speed of 6 mm/s, followed by gas quenching by N_2 stream under a pressure of 20 psi. The films were transferred to a preheated hotplate at 100 °C immediately for 10 minutes of annealing. $PDAI_2$ was post-deposited onto perovskite by blade coating with a speed of 5 mm/s. Then, the perovskite films were transferred into a vacuum chamber for thermal evaporation. C_{60} (23 nm) and BCP (7 nm) were sequentially deposited on the perovskite films at a vacuum pressure of 3×10^{-6} mbar. Cu (100 nm) was finally evaporated under a shadow mask at a vacuum pressure of 3×10^{-6} mbar as the top electrode. Devices were encapsulated by using cover glass and UV-curable epoxy for stability measurements.

Fabrication for WBG PSMs

Major steps are the same as the cell fabrication except following things. For module fabrication, SnO_x was deposited by atomic layer deposition (ALD) to replace BCP for better resistance to the environment during P2 scribes. SnO_x was deposited at relative low temperature (100 °C) by periodic pulse of tetrakis(dimethylamino) tin(IV) and deionized water for 200 cycles. 120 nm of Cu was thermally evaporated without a shadow mask as the top electrode. Laser scribing was performed on a True Mark station 5000, using a nanosecond pulsed Nd:YVO4 laser with a wavelength of 355 nm. P1 scribes were done on indium tin oxide (ITO) glass substrates before HTL deposition. P2 scribes were performed after SnO_x deposition. P3 scribes were done after Cu deposition. The modules fabricated on the 5 × 5 cm^2 substrates contain 6 cells with a width of 6 mm for each cell. The length of the cell is defined as 30 mm by additional scribes after P3 scribes. The defined area of WBG PSMs is 10.8 cm^2.

Fabrication for all-perovskite tandem cells and mini-modules

All the procedures of the fabrication of flexible all-perovskite TSCs are the same as the flexible WBG PSCs until completing C_{60} deposition. Samples were then transferred to ALD chamber, and SnO_x was deposited at relative low temperature (100 °C) by periodic pulse of tetrakis(dimethylamino) tin (IV) and deionized water for 200 cycles. 1.2 nm gold (Au) cluster was then deposited onto SnO_x by thermal evaporation at ultra-slow deposition rate of 0.02 Å/s. PEDOT:PSS diluted with IPA (volume ratio of 1:2) was spin-coated onto Au cluster at 4000 rpm for 50 s, followed by annealing at 100 °C for 5 min. The samples were them transferred into the glovebox to finish the fabrication of NBG Sn-Pb bottom subcell. NBG perovskite ink was blade-coated onto PEDOT:PSS at a coating speed of 1 mm/s, followed

by gas quenching at 30 psi. $EDAI_2$ was post-deposited onto perovskite by blade coating with a speed of 5 mm/s. The films were immediately transferred to a 100 °C hotplate for 10 minutes of annealing. Then, the perovskite films were transferred into a vacuum chamber for thermal evaporation. C_{60} (23 nm) and BCP (7 nm) were sequentially deposited on the NBG perovskite films at a vacuum pressure of 3 × 10^{-6} mbar. Cu (100 nm) was finally evaporated under a shadow mask at a vacuum pressure of 3 × 10^{-6} mbar as the top electrode.

Fabrication of all-perovskite tandem solar modules is very similar to that of flexible WBG perovskite solar modules, but the following points should be noted. In top subcell, SnO_x was used instead of BCP. P2 scribes were performed after SnO_x deposition of the top subcell. Then extra SnO_x layer (100 cycles) was deposited after P2 scribes to work as a diffusion barrier.

Device and film characterization

J-V measurements:

Cells/modules were tested under standard AM1.5 G illumination and are measured using a Keithley 2400 source meter under four-contact mode. A certified monocrystalline silicon solar cell (RS-ID-5, Fraunhofer-ISE) is used to calibrate a class ABA solar simulator (LOT-QuantumDesign) to 1 sun AM1.5 G illumination. For carrying out the steady-state efficiency measurement, an MPP tracking system with the perturb and observe (P&O) method is employed. The shadow mask has an area of 0.057 cm^2.

External quantum efficiency (EQE):

The device EQE was measured with a lock-in amplifier. The probing beam is generated by a chopped white source (900 W, halogen lamp, 280 Hz) and a dual grating monochromator. A certified single-crystalline silicon solar cell is used as a reference cell.

Operational stability test:

Operational stabilities of solar cells were performed using Litos Lite platform (https://www.fluxim.com/litos-lite) equipped with a temperature-controlled sample box. Testing samples were heated up to 65 ˚C before stressing under maximum-power-point (MPP) to meet ISOS-L-2 protocol.

Contact angle measurements:

Contact angle measurements were carried out on a drop shape analyzer (Krüss, DSA30E) with Laplace equation. Approximately 10 μL of WBG perovskite ink was dropped onto the substrate through a syringe needle, and images were captured within 10 seconds.

X-ray Photoelectron Spectroscopy (XPS):

XPS was performed with a PHI Quantum 2000 equipped with a monochromatic Al Kα source (1486.6 eV). High-resolution spectra were recorded with an energy step size of 0.2 eV and a pass energy of 23.50 eV. Elemental peak identification was done using CasaXPS software. All XPS spectra were corrected for charge effects by setting the C1s binding energy to 284.8 eV.

Surface-enhanced Raman spectroscopy (SERS):

1 nm gold cluster was thermally evaporated onto the Me-4PACz film to amplify its Raman response. Raman measurements were performed using a WITec alpha300 Raman instrument. The sample was excited with a 488 nm laser using a Zeiss LD EC Epiplan-Neofluar DIC 50× objective with a numerical aperture of 0.55. The absolute laser power at the sample was 0.5 mW, corresponding to an estimated power density of approximately 0.54 mW μm^{-2} assuming a diffraction-limited laser spot. Spectra were collected using a 600 grooves mm^{-1} grating blazed at 500 nm. Raman mapping was performed over a 50 × 50 μm^2 area using an 80 × 80 pixel grid, with an integration time of 20 s per spectrum, resulting into 6400 spectra per map. The individual spectra acquired from the Raman map were subsequently averaged to obtain a representative spectrum of the measured area.

Atomic force microscopy (AFM) and Kelvin probe force microscopy (KPFM):

AFM and KPFM measurements were performed using a Flex-AFM system (Nanosurf, Switzerland), which was equipped with a C3000 controller and a Zurich Instruments HF2 lock-in amplifier. To prevent sample degradation under ambient conditions, all measurements were conducted inside a nitrogen-filled glovebox with O_2 and H_2O concentrations below 0.1 ppm. The setup was placed on a vibration-isolation table to minimize mechanical noise during imaging.

KPFM measurements were carried out in amplitude-modulation mode (AM-KPFM) using a single-pass approach at the second resonance frequency of the cantilever.[1] Conductive Pt/Ir-coated PPP-NCLPt tips from Nanosensors with a nominal spring constant of 30 N/m and a resonance frequency of f_1 = 160 kHz were used; the tip was electrically grounded throughout all measurements. An AC modulation voltage (amplitude: 0.5-1 V; frequency: ~1 MHz, tuned to the second resonance frequency of the cantilever) was applied to the sample, generating an oscillating electrostatic force that was detected by the HF2 lock-in amplifier. Simultaneously, a feedback-controlled DC bias was applied to the sample to nullify this force component, yielding the local contact potential difference (CPD) between the tip and the sample. The work function (WF) of the sample was subsequently derived from the CPD using the following equation: $WF_{sample} = WF_{tip} + CPD$.

Work function calibration was performed using freshly cleaved, highly oriented pyrolytic graphite (HOPG) as a reference with an established work function of 4.5 eV.[2] The tip work function was determined from HOPG measurements taken immediately before or after imaging the sample, in order to account for contamination or wear of the tip. Topography images were acquired in dynamic mode (AM-AFM) at a scan rate of 1-2 s/line with a setpoint

amplitude of ~80% (corresponding to an approximate amplitude of 10 nm) of the free oscillation amplitude. Image processing and analysis were performed using Gwyddion, with plane subtraction and line-by-line flattening applied as baseline corrections to the topography channel. The CPD shows the raw data. The CPD range, defined as the difference between the maximum and minimum CPD values across the mapped area, was used as a quantitative measure of lateral surface potential homogeneity.

Time resolved photoluminescence (TRPL):

TRPL was measured using the MicroTime 100 system from PicoQuant. A 639 nm pulsed laser diode (pulse width at ~100 ps) was used to activate samples.

Photoluminescence quantum yield (PLQY):

PLQY was measured using LuQY Pro system from Quantum Yield Berlin. 1-sun equivalent laser with a wavelength of 532 nm was used activate samples at spot size of 0.1 cm^2. PLQY was obtained by taking the average from 5 measurements at the same spots.

X-ray fluorescence (XRF) mapping:

Elemental distribution maps were obtained using a Fischerscope XDV-SDD X-ray fluorescence instrument equipped with a programmable XY stage and Z-axis for automated measurements. The X-ray source consists of a microfocus tube with a rhodium target and a beryllium window. Detection was performed using a Peltier-cooled silicon drift detector (SDD) with an effective area of 50 mm^2. Samples were positioned beneath the device optics using the integrated stage motors. A beam diameter of 1 mm was used. Measurement points were defined on a regular grid across the sample surface. For each position, elemental intensities were extracted from the characteristic emission lines following background subtraction. Film thickness was additionally estimated from the attenuation of substrate-related signals.

X-ray diffraction (XRD) mapping:

Spatially resolved X-ray diffraction (XRD) measurements were conducted in a PANalytical Empyrean Multicore diffractometer using Cu Ka radiation (λ = 1.5418 Å) in Bragg-Brentano geometry. A grid of acquisition points was predefined, and the sample was moved within the diffractometer using the integrated x-y stage. At each position, diffraction patterns were collected over 12°-15° 2 theta range, with a step size of 0.013° and a beam size of 2 mm x 2 mm.

Scanning electron microscope (SEM):

Top-view SEM images were taken with a Hitachi S-4800 using 5 kV acceleration voltage. Image J software was used to count grain numbers and their sizes in SEM images. The cross-section SEM images were obtained with a Helios 660 (Thermo Fischer Scientific) focused ion beam (FIB)-SEM dual beam microscope. A Pt capping layer was applied to protect the sample during the ion-beam cut and to improve the cross-section quality. The cross-section was milled with a Ga-ion beam at 30 kV accelerating voltage, starting with a

current of 9 nA, followed by successive cleaning cuts at 80 pA. The SEM images were taken with an acceleration voltage of 2 kV and a current of 0.2 nA for the electron beam. The detector chosen is the Through-Lens Detector in secondary electrons mode.

PLQY imaging:

PL measurements were performed using the solar cell multi-imaging tool Vitios (Fluxim AG, Switzerland) equipped with a Basler ace 2 camera with spectral sensitivity between 400 and 1000 nm.[3] The camera is equipped with a Fujinon lens and a 675 nm long-pass filter. The light source is a monochromatic 405 nm LED array. The raw photoluminescence image of the perovskite film is acquired in grayscale. Conversion of the image into a PLQY map requires a photoluminescence measurement of a white reference. Following the procedure proposed by Dasgupta et al., $BaSO_4$ was used as the white reference, owing to its Lambertian reflection profile and near-unity reflectivity. Two assumptions underlie this approach: (i) all incident photons are absorbed by the sample, and (ii) the PL emission profile of the sample is similar to the reflection profile of $BaSO_4$, so that the white reference provides an estimate of the local incident photon flux. Under these assumptions, the PLQY is obtained as:

$$PLQY = \frac{\Phi_{SC,total}}{\Phi_{ref,total}} = \frac{S_{SC} \cdot \tau_{int,ref} \cdot \int QE(\lambda_{LED}) \cdot \Phi_{ref} d\lambda}{S_{ref} \cdot \tau_{int,SC} \cdot \int T(\lambda_{PL}) \cdot QE(\lambda_{PL}) \cdot \Phi_{SC} d\lambda}$$

, where S, τ_{int}, QE, Φ_{ref}, Φ_{SC}, T, and λ represent signal (solar cell/ reference), integration time (Exposure time), quantum efficiency camera, photon flux reference (LED spectrum), photon flux solar cell (PL spectrum), filter transmission, and photon wavelength, respectively.

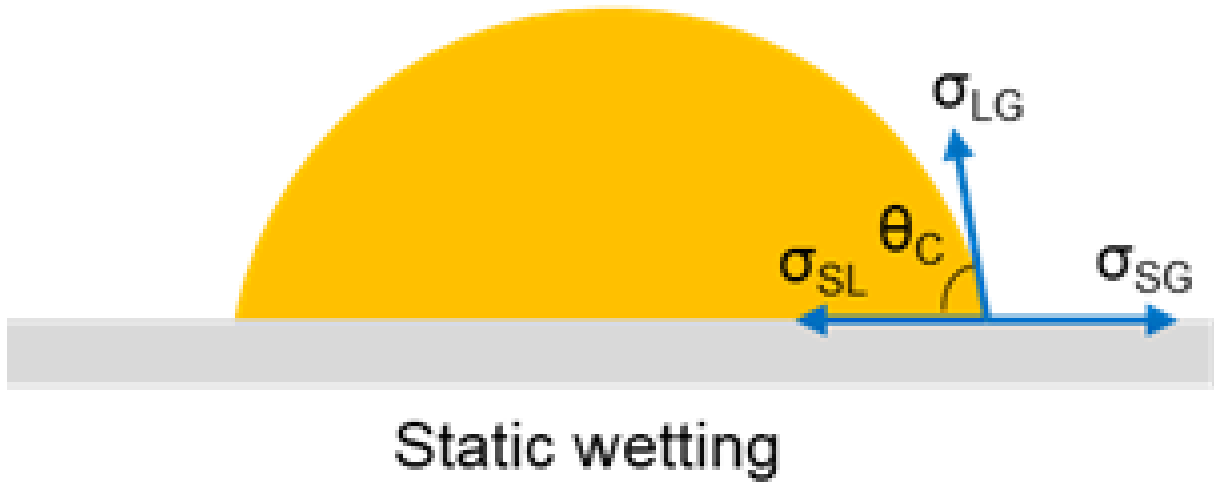

$$\sigma_{LG}\cos(\pi-\theta_C)+\sigma_{SL}+\sigma_{SG}=0$$

Figure S1: Schematic diagram of static wetting and contact angle.

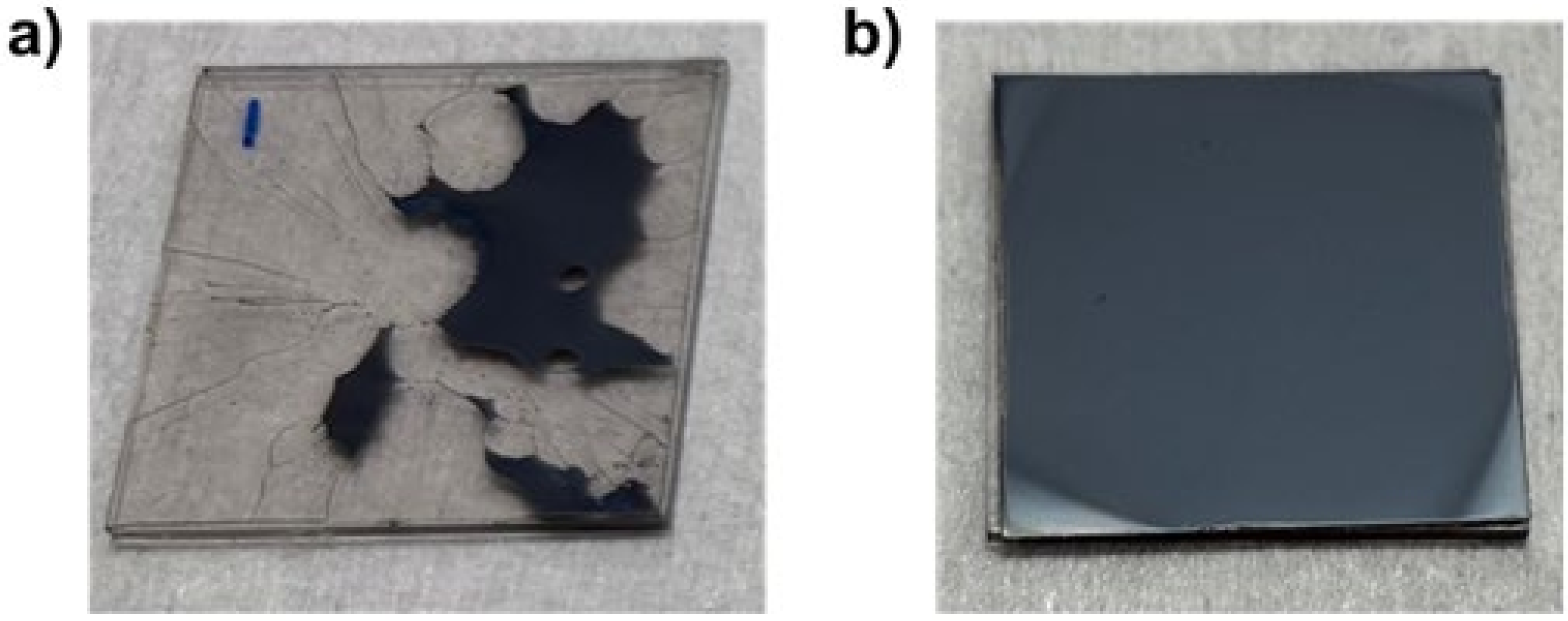

Figure S2: Optical photos of WBG perovskite films spin-coated on **a)** PEN/ITO/Me-4PACz and **b)** PEN/ITO/NiO_x/Me-4PACz.

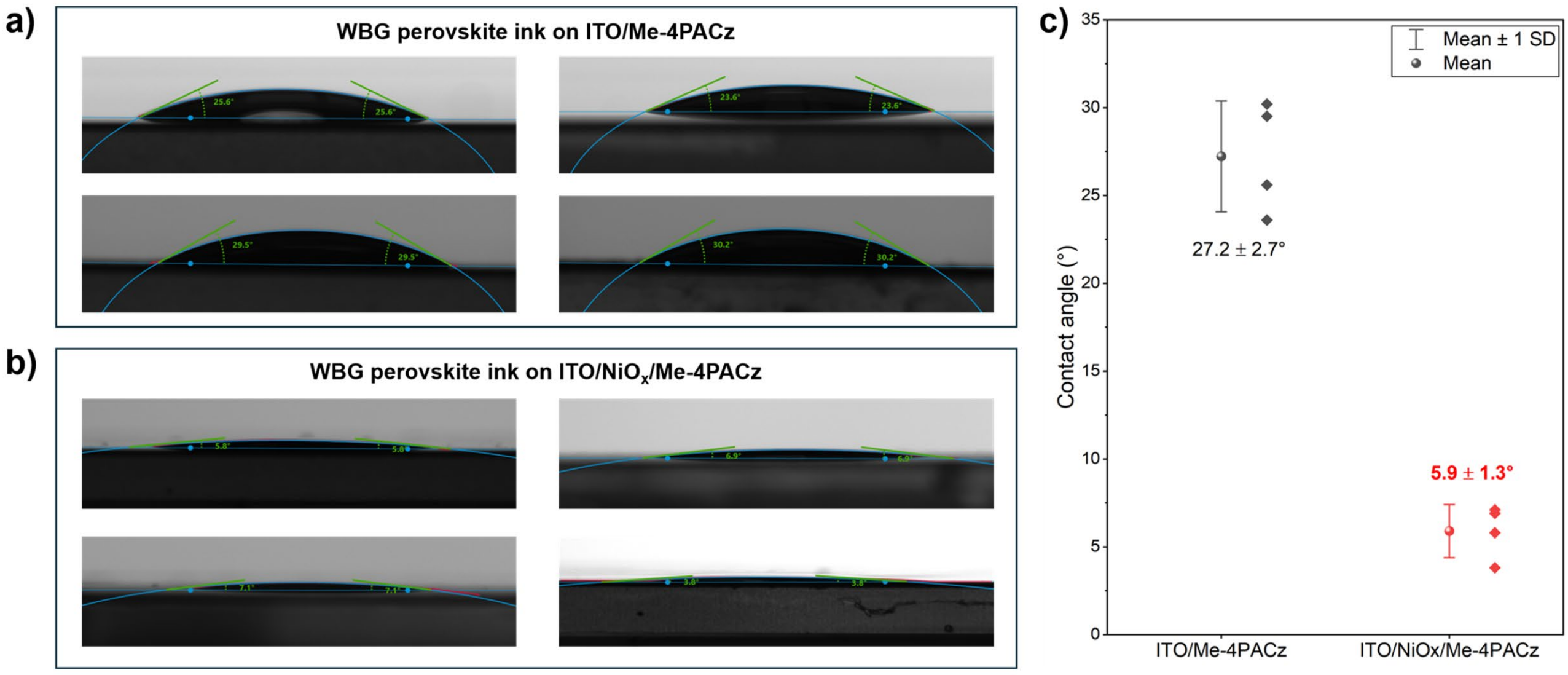

Figure S3: Figure R1 (Figure S3): **a-b)** Microscopic images of perovskite ink droplets on **a)** ITO/Me-4PACz and **b)** ITO/NiO_x/Me-4PACz substrates. **c)** Statistical comparison of the contact angles of perovskite ink on ITO/Me-4PACz and ITO/NiO_x/Me-4PACz substrates.

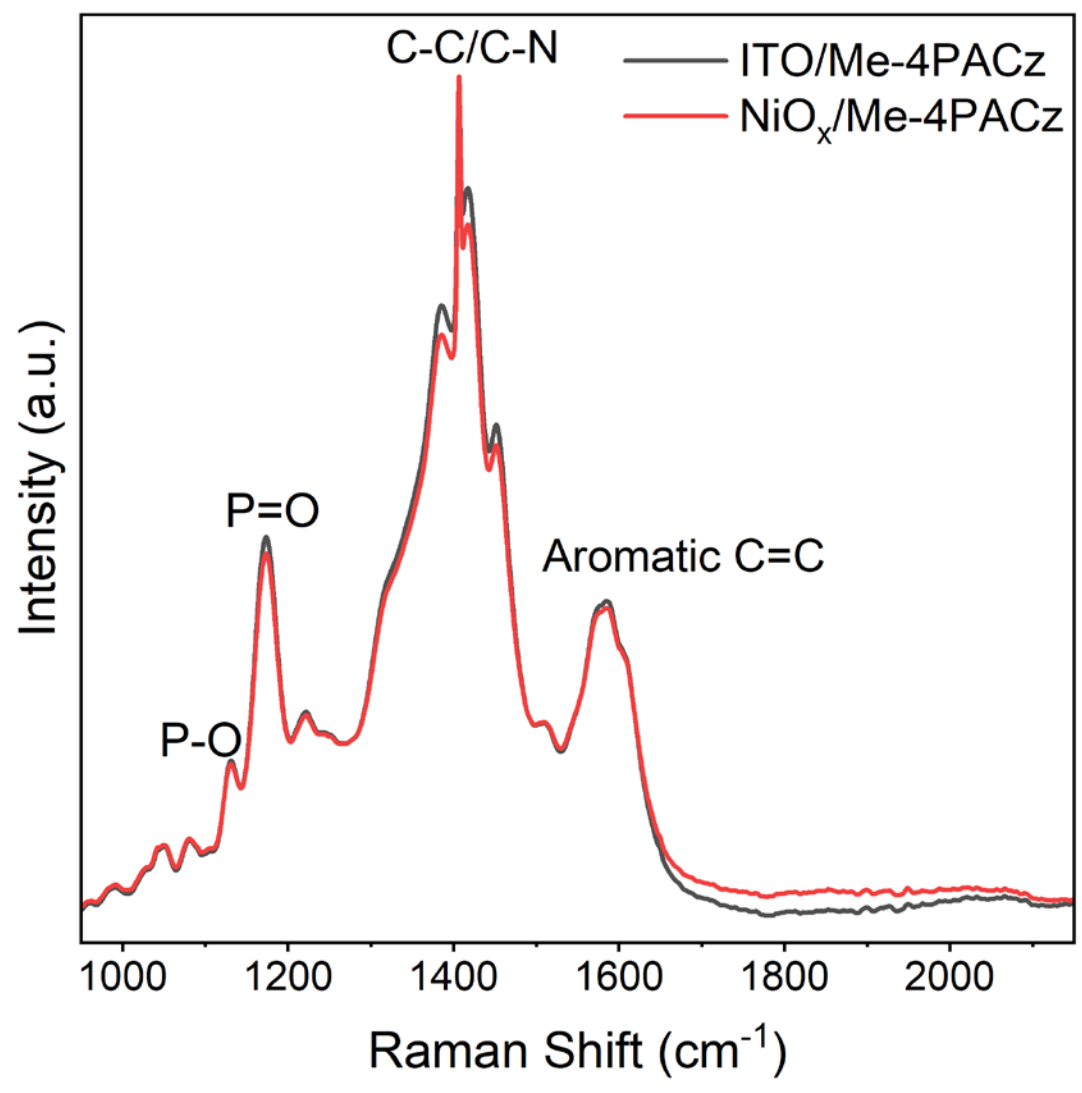

Figure S4: SERS spectra of Me-4PACz coated ITO and ITO/NiO_x.

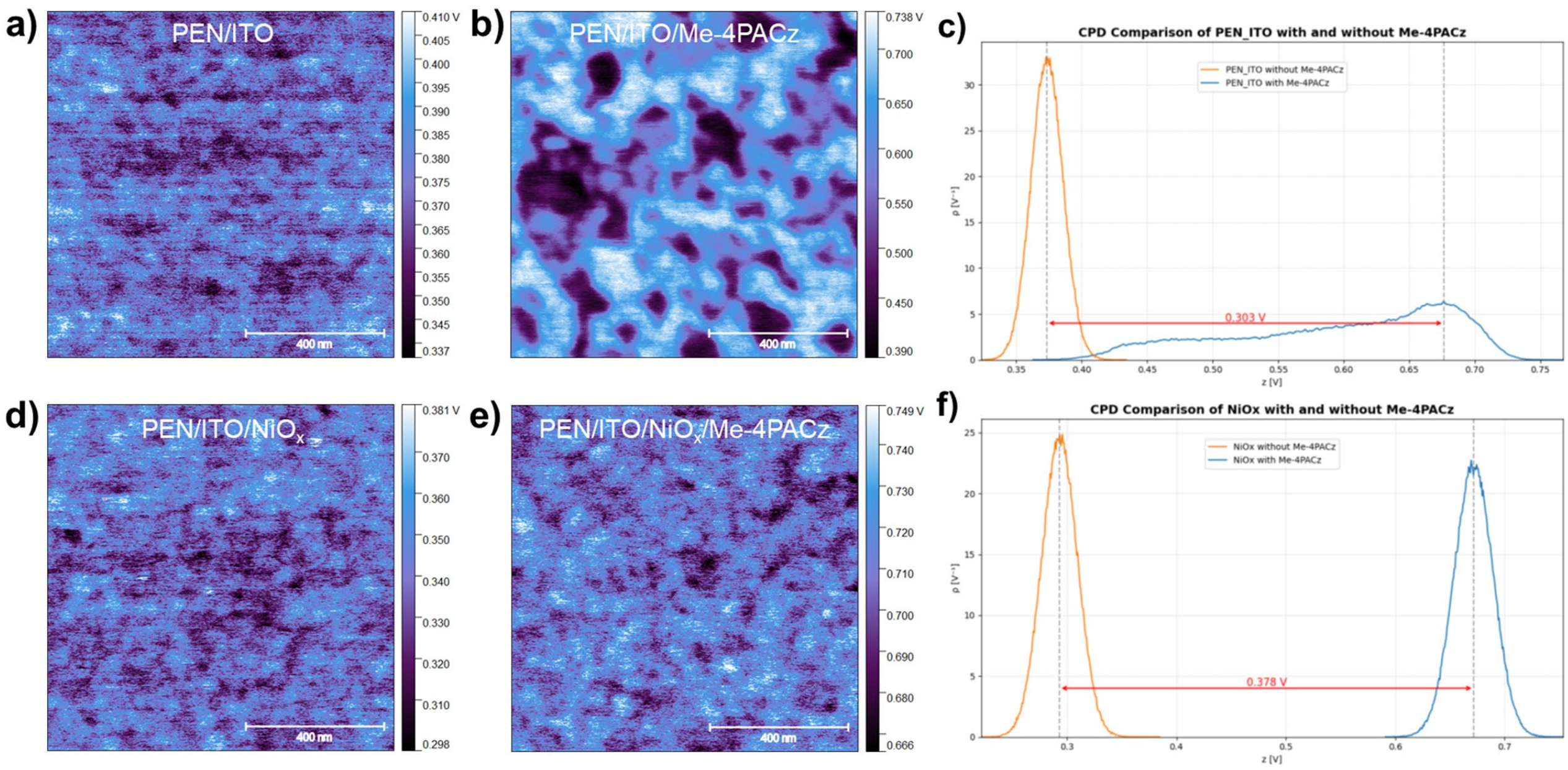

Figure S5: KPFM images of **a)** bare ITO, **b)** Me-4PACz-coated ITO, **d)** bare ITO/NiO_x, and **e)** Me-4PACz-coated ITO/NiO_x. Comparison of CPD for **c)** ITO and **f)** ITO/NiO_x films before and after Me-4PACz coating. The scale bars in **a-d)** are 400 nm.

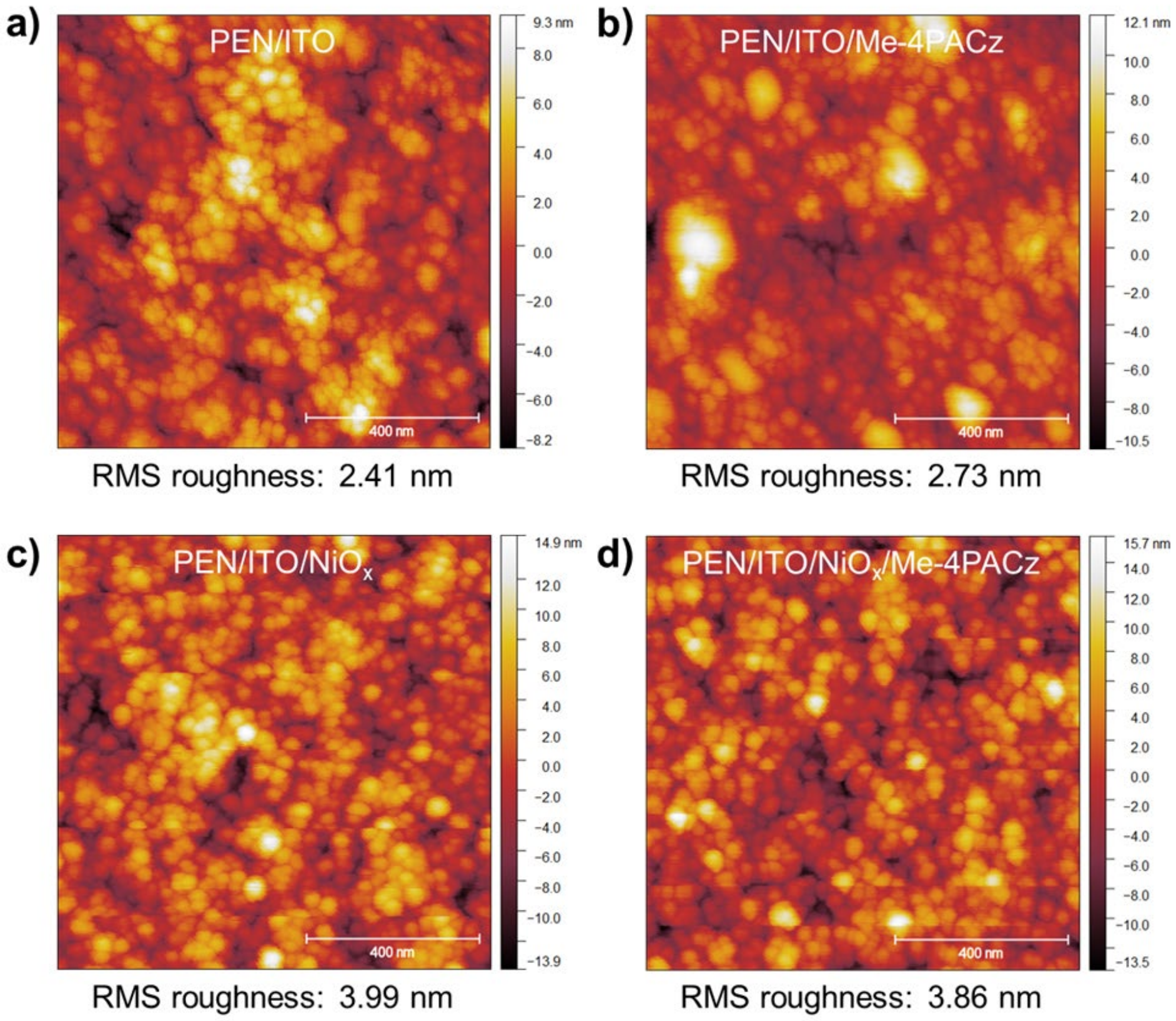

Figure S6: AFM images of **a)** bare ITO, **b)** Me-4PACz-coated ITO, **c)** bare ITO/NiO_x, and **d)** Me-4PACz-coated ITO/NiO_x. The scale bar is 400 nm.

Note S1: Crystallinity of 4PADCB and its effect on perovskite wetting

We performed contact angle and XRD measurements on 4PADCB-based samples to confirm that inserting NiO_x interlayer is also applicable to 4PADCB to achieve improved wetting for perovskite ink. As shown in **Figure S7**, 4PADCB deposited onto NiO_x shows poorer crystallinity than that on ITO, indicating the suppressed 4PADCB aggregation after introducing NiO_x interlayer. As a result, the wettability of WBG perovskite ink on 4PADCB is greatly improved after introducing NiO_x interlayer, with a reduction of contact angle from 18.4° to 6.0° (**Figure S8**). Owing to the improved wettability, we are able to obtain uniform coating of WBG perovskite films with excellent coverage on flexible substrates by doctor-blade coating.

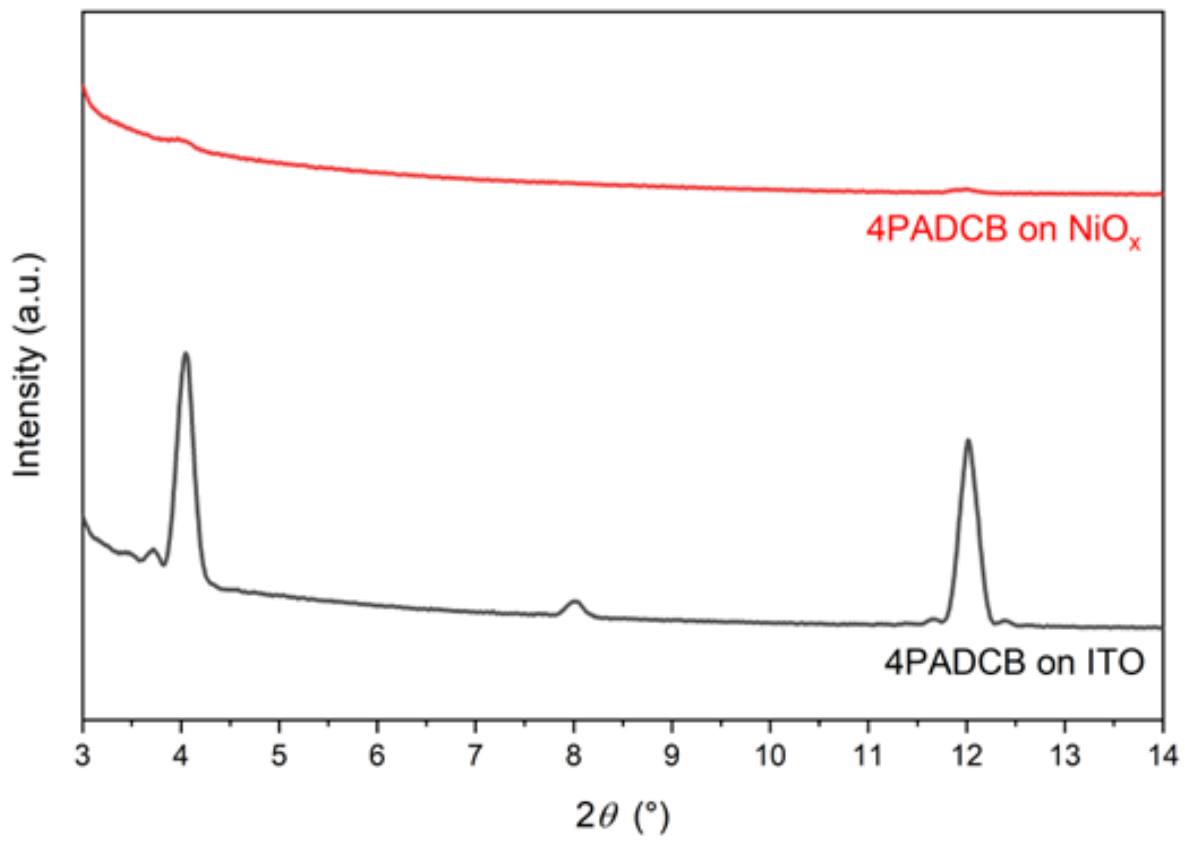

Figure S7: XRD patterns of 4PADCB films coated on ITO and NiO_x. Saturated 4PADCB ink is used to prepare 4PADCB films.

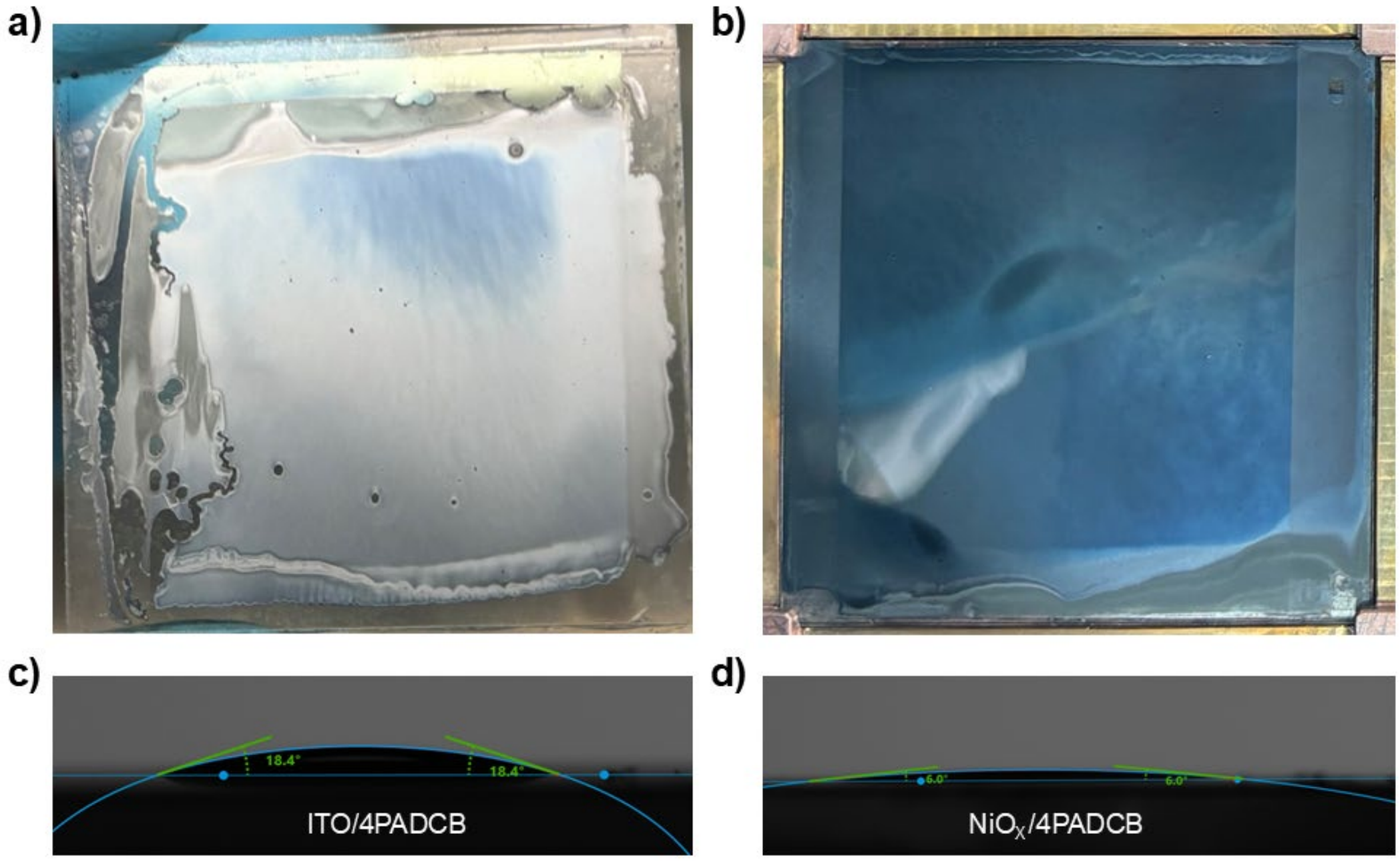

Figure S8: Photographs of WBG perovskites coated on **a)** ITO/4PADCB and **b)** ITO/NiO_x/4PADCB. Contact angle of perovskite ink on **c)** ITO/4PADCB and **d)** ITO/NiO_x/4PADCB.

Note S2: SAM screening to obtain better HTL/perovskite interface

To evaluate the effect of these SAMs on charge-carrier dynamics at the buried interface, time-resolved photoluminescence (TRPL) measurements were performed, with excitation from the SAM side. The resulting decay curves are shown in **Figure S9a**. The initial fast decay is dominated by hole extraction from the perovskite to the HTL, while the slower component is associated with interfacial recombination.[4] We fitted TRPL results with a bi-exponential function and then took the time-dependent derivative of the logarithm of the fitted decay to extract differential lifetime, i.e., $\tau_{\text{diff}}(t) = -[d(\ln I(t))/dt]^{-1}$. (**Figure S9b**). The fitting results are summarized in **Table S1**. Among the studied SAMs, the MeO-2PACz-based perovskite film exhibited the shortest τ_1 (65.14 ns), indicating the most efficient hole extraction, followed by Me-4PACz (70.46 ns), 4PADCB (79.88 ns), and 2PACz (118.32 ns). Regarding interfacial recombination, τ_2 values for 2PACz, Me-4PACz, and 4PADCB films were comparable (~390 ns), all significantly longer than that of MeO-2PACz (269.21 ns), suggesting more pronounced recombination in the latter. The observed variations in TRPL lifetimes (τ_1 and τ_2) among different SAMs are attributed to their distinct dipole moments, which modulate the work function of the underlying NiO_x in different extent.[5-6] A deeper work function can impose an energy barrier for hole extraction (longer τ_1), while a shallower work function may increase the interfacial recombination (shorter τ_2) due to inefficient energetic alignment between NiO_x and the perovskite.

Based on the TRPL results, Me-4PACz and 4PADCB were selected for further investigation. Absolute photoluminescence (PL) spectroscopy was performed at five different locations across flexible 5 × 5 cm^2 perovskite films coated on Me-4PACz and 4PADCB to evaluate their PL quantum yield (PLQY) (**Figure S10**). The Me-4PACz-based perovskite film exhibited a higher average PLQY of 0.25%, compared to 0.14% for the 4PADCB-based film, indicating better radiative efficiency and lower non-radiative recombination. Consequently, Me-4PACz was chosen as SAM to pair with NiO_x for subsequent film and device fabrication.

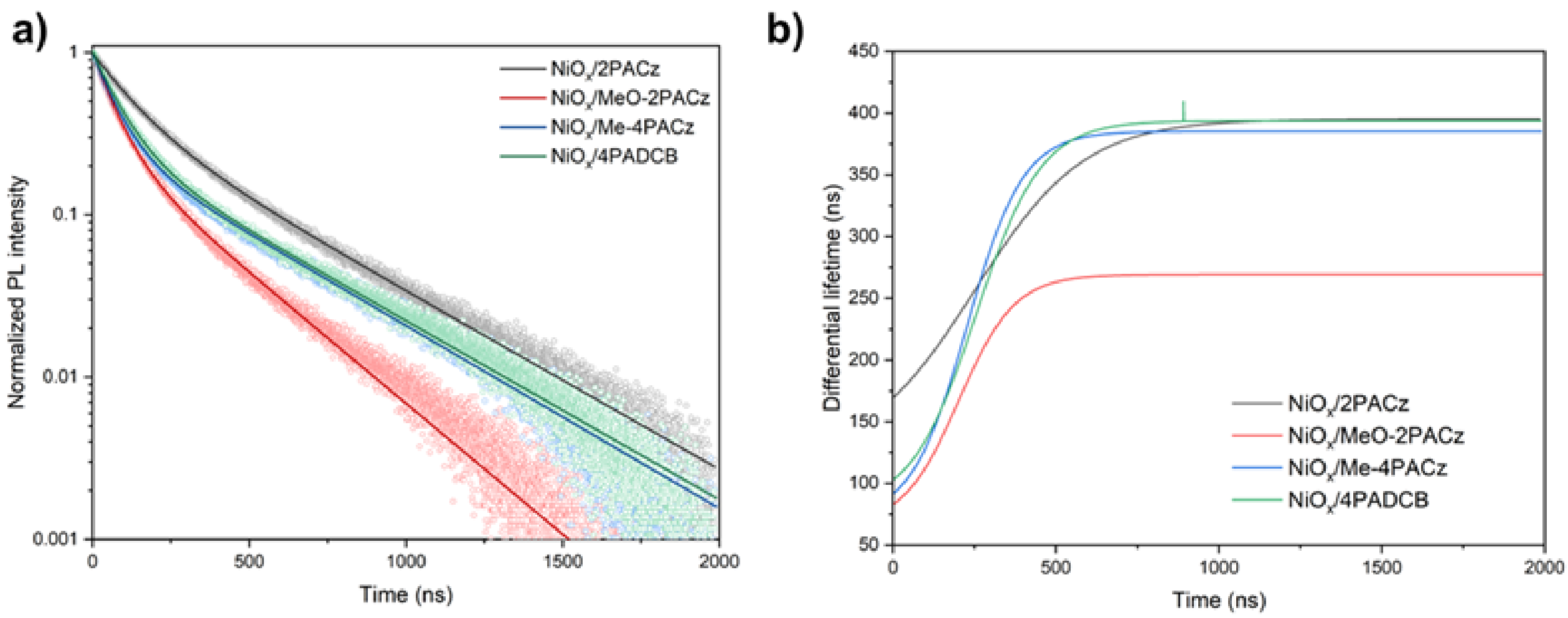

Figure S9: **a**) TRPL decay curves and **b)** computed differential lifetimes of WBG perovskite films coated on NiO_x/SAM with bi-exponential fittings.

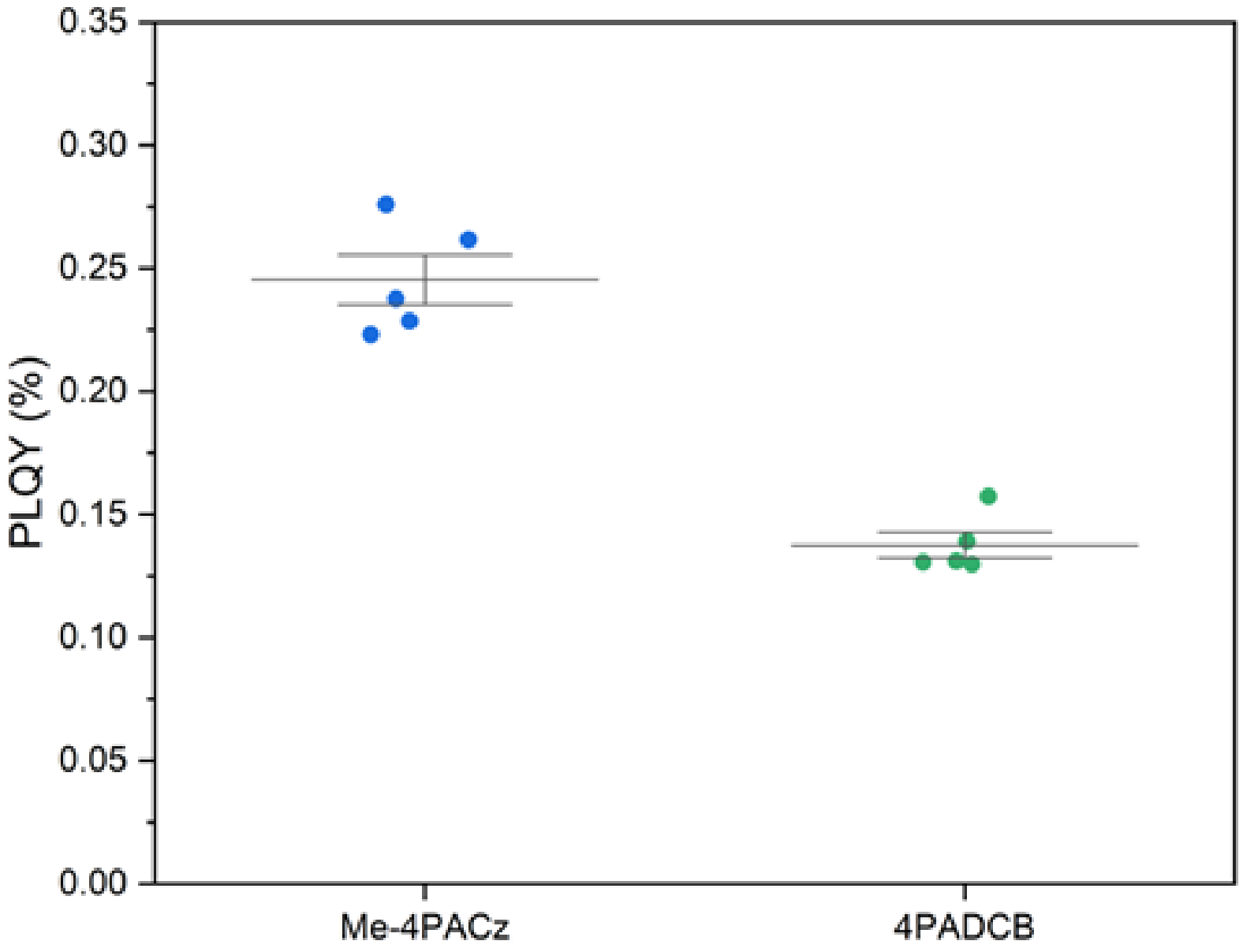

Figure S10: Statistical PLQY value of WBG perovskite at five spots across the 5 × 5 cm^2 flexible WBG perovskite films.

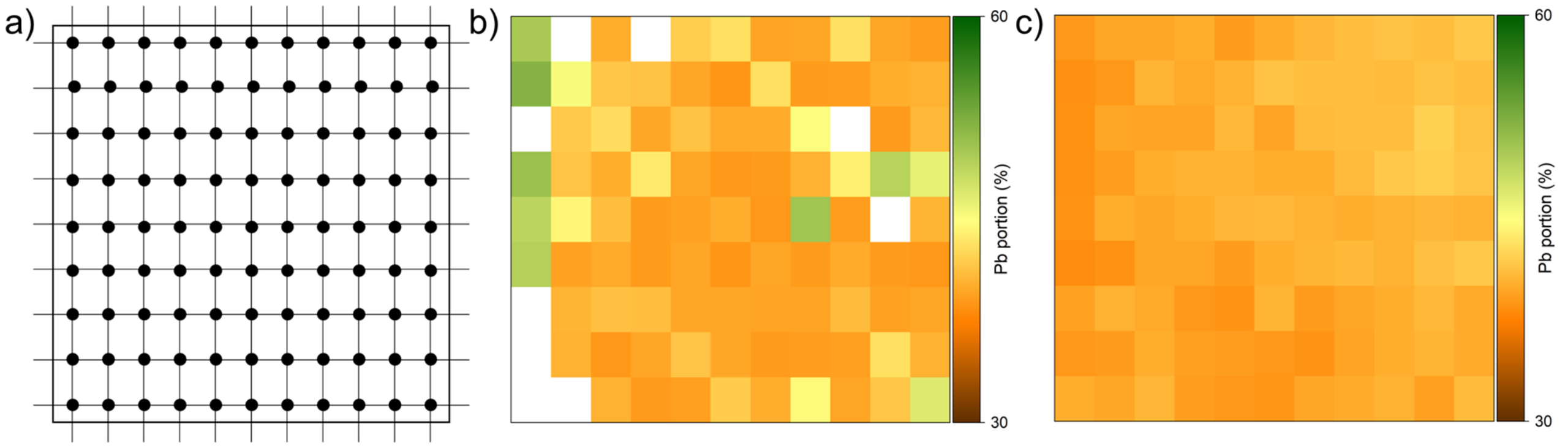

Figure S11: **a**) Location map of test points in XRF mapping. Heatmaps of Pb portion in the total amount of Pb, Cs, I, and Br in **b)** reference and **c)** NiO_x/Me-4PACz-based 5 × 5 cm^2 WBG perovskite films.

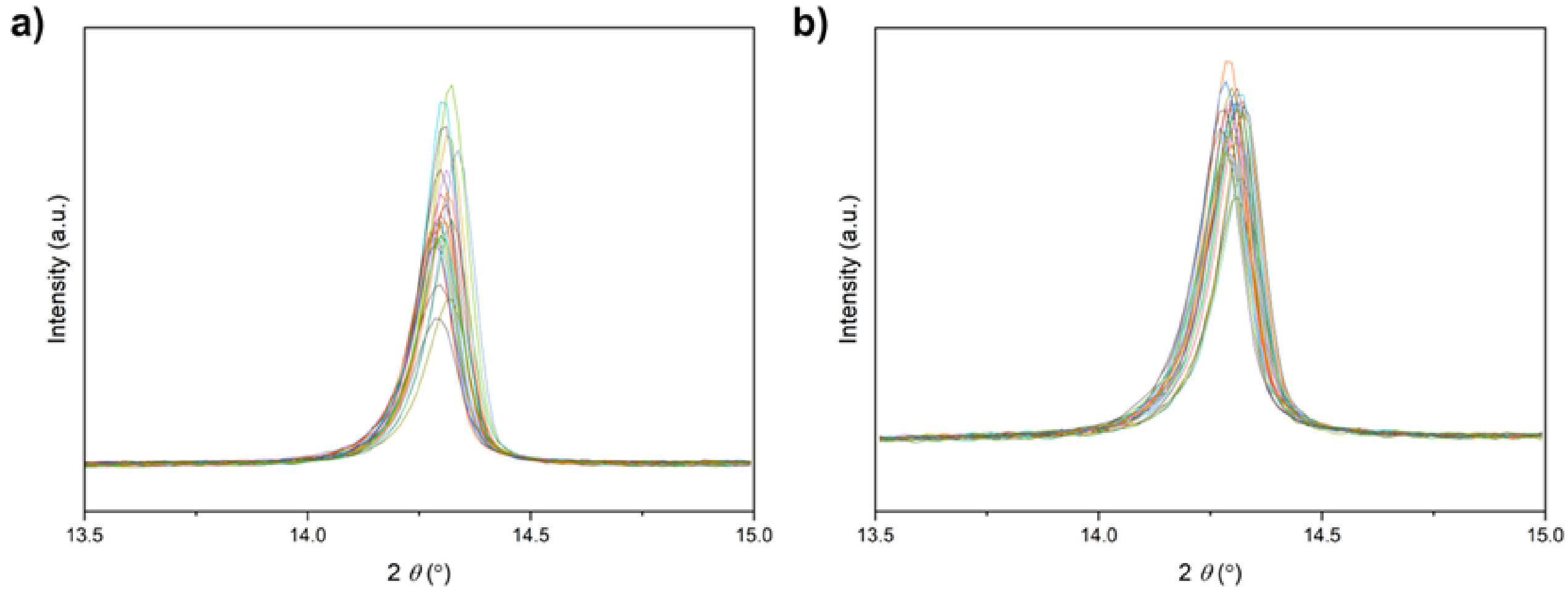

Figure S12: XRD patterns of **a)** reference and **b)** NiO_x/Me-4PACz-based perovskite films taken from 25 spots across different spatial locations.

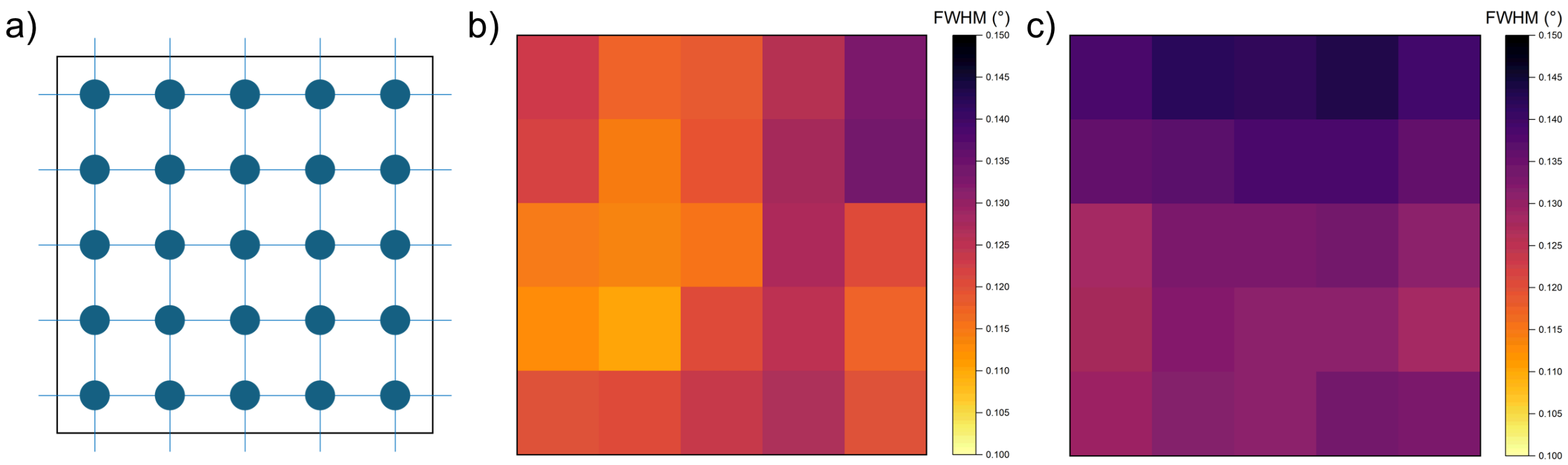

Figure S13: a) Location map of test points in XRD mapping. Heatmaps of FWHM in the perovskite (100) diffraction peak obtained from XRD mapping measurements on **b)** reference and **c)** NiO_x/Me-4PACz-based 5 × 5 cm² flexible WBG perovskite films.

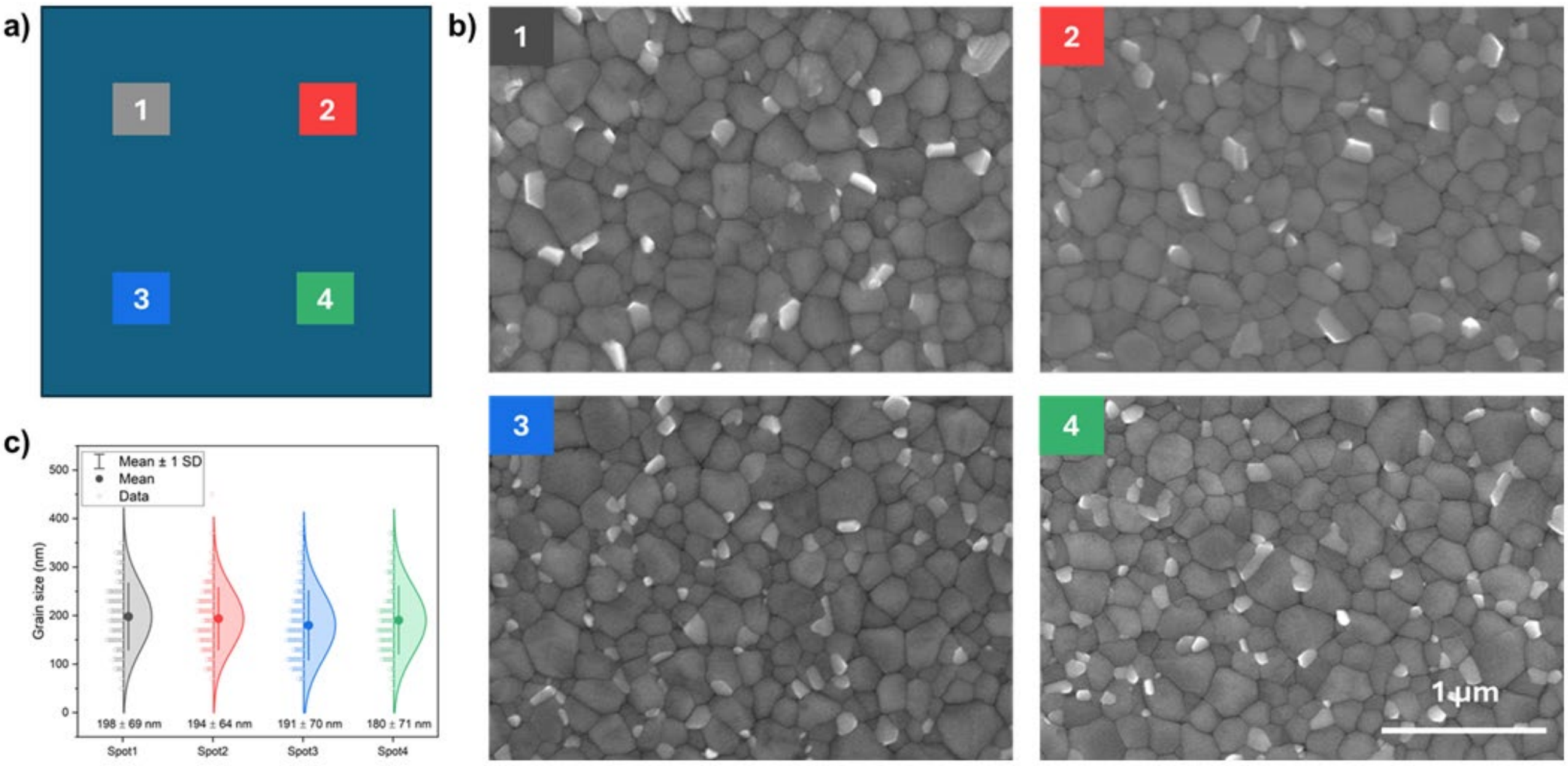

Figure S14: a) Location map of test points for SEM. **b)** SEM images of NiO_x/Me-4PACz-based perovskite films taken from 4 different spots across 5 × 5 cm² flexible WBG perovskite films. Scale bar:1 μm. **c)** Distribution of grain sizes shown in SEM images.

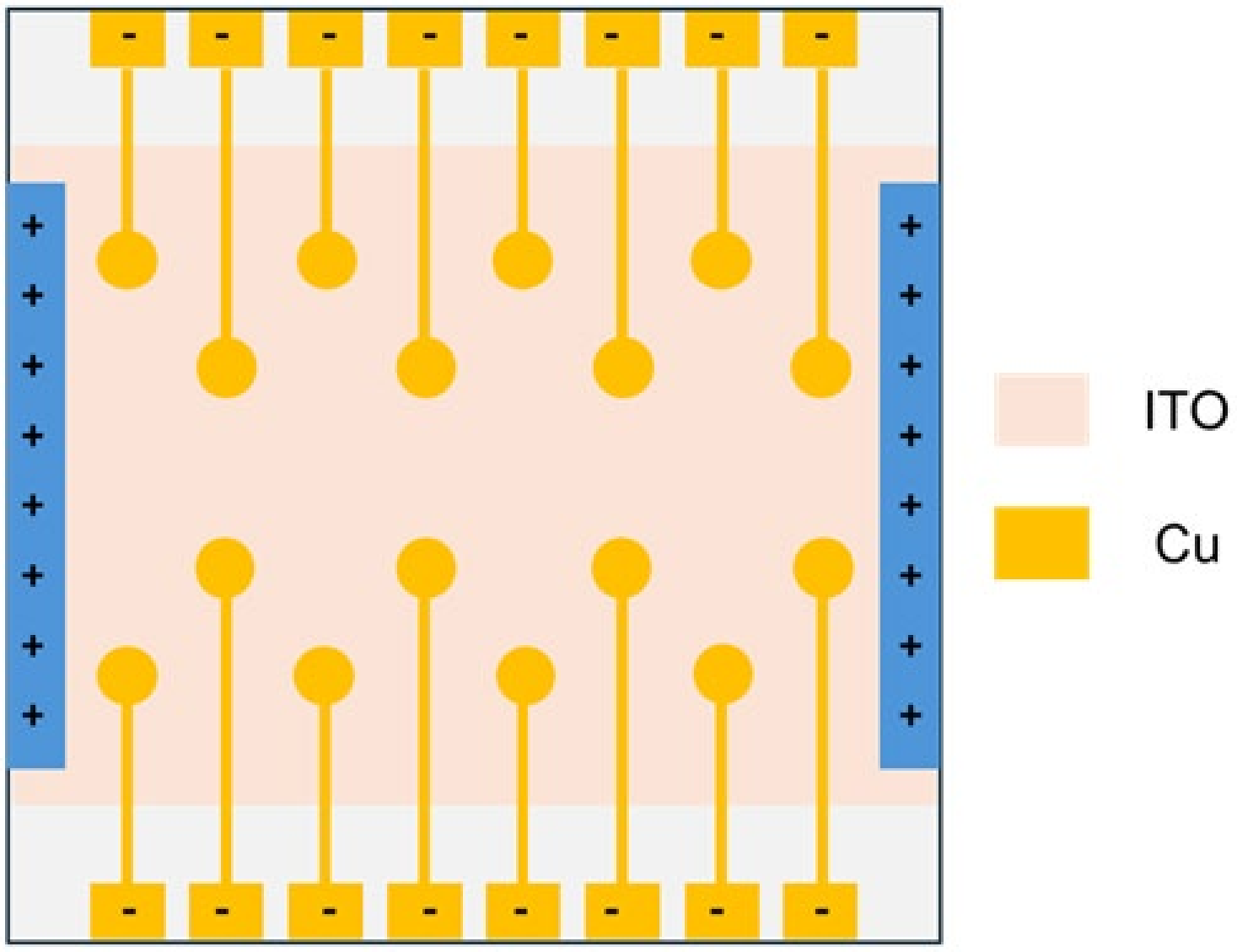

Figure S15: Schematic pattern of flexible WBG PSCs fabricated on 5 × 5 cm^2 substrates, containing 16 pixels.

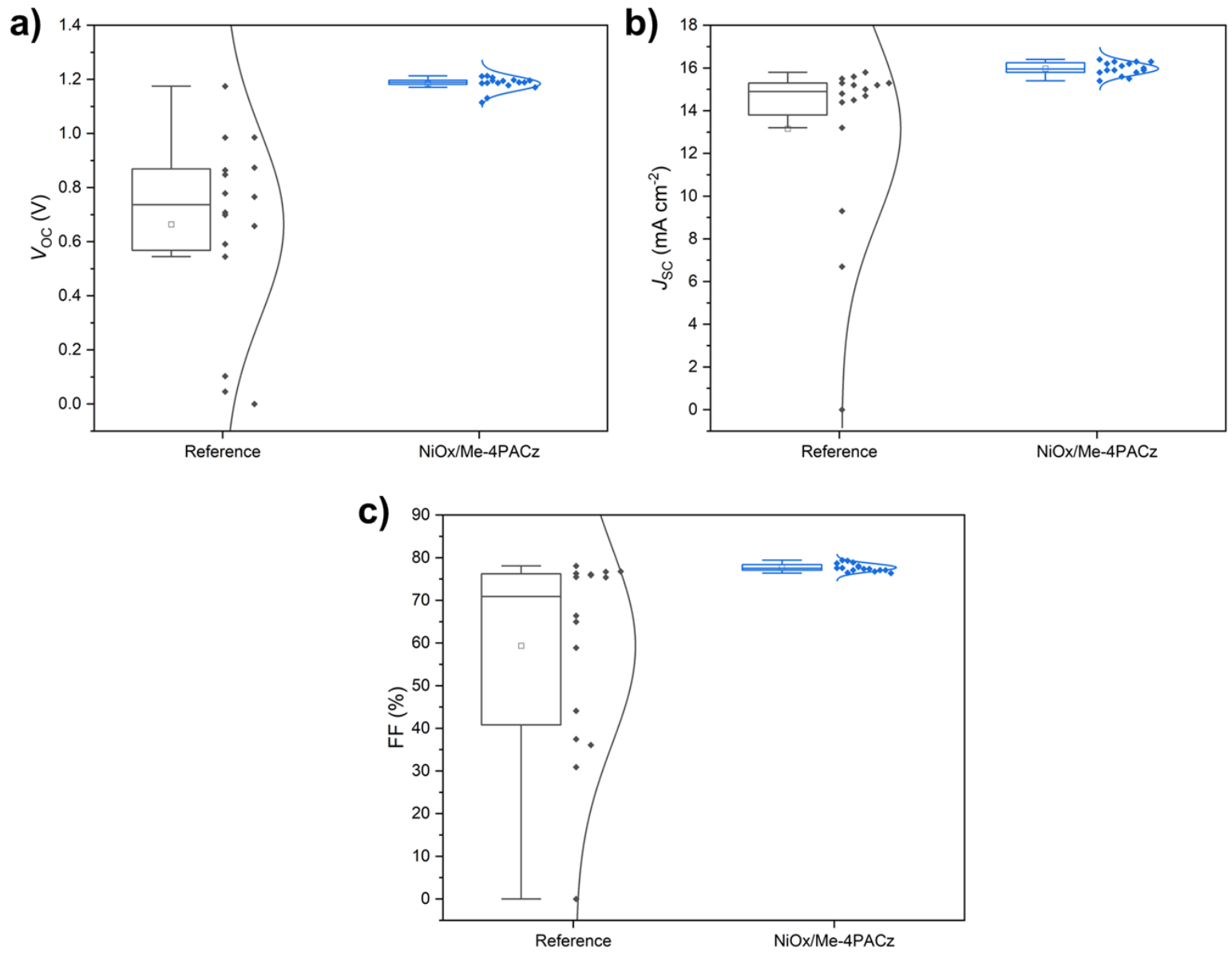

Figure S16: Statistical PV parameters of reference and NiO_x/Me-4PACz-based flexible WBG PSCs. **a)** V_{OC}, **b)** J_{SC}, and **c)** FF.

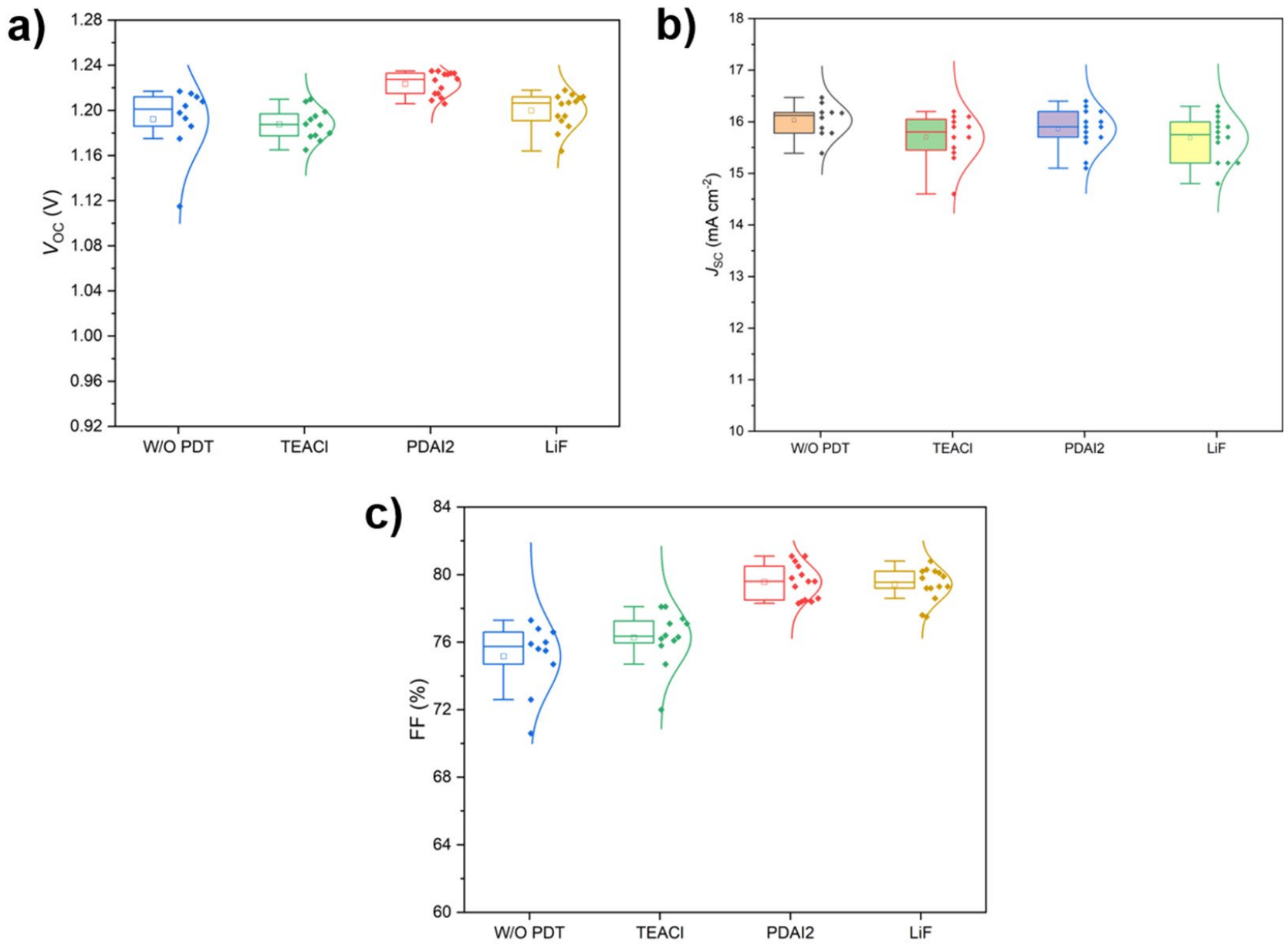

Figure S17: Statistical PV parameters of flexible WBG PSCs with different PDT strategies. **a)** V_{OC}, **b)** J_{SC}, and **c)** FF.

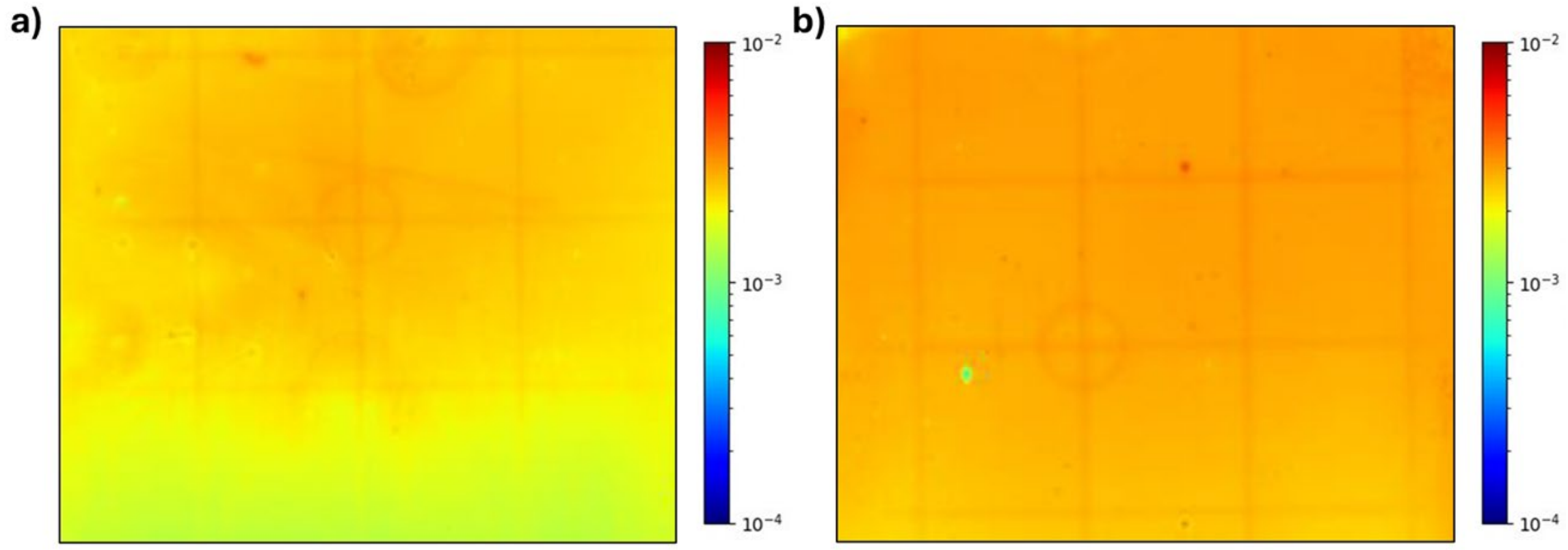

Figure S18: PLQY imaging of flexible WBG perovskite films **a)** before and **b)** after $PDAI_2$ PDT. The grids on image are from the plate where the sample was positioned.

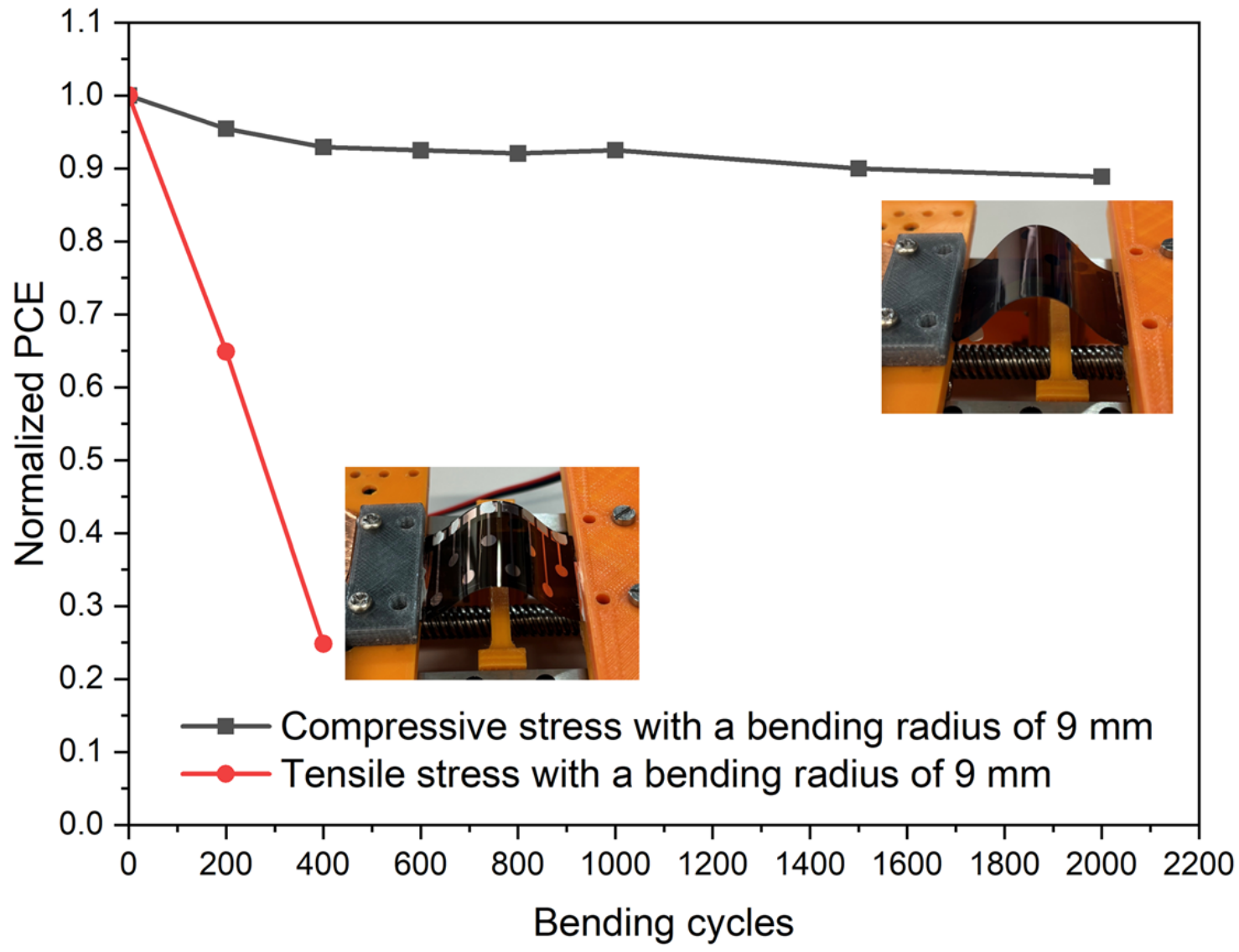

Figure S19: Mechanical stability of unencapsulated flexible WBG PSCs at a bending radius of 9 mm.

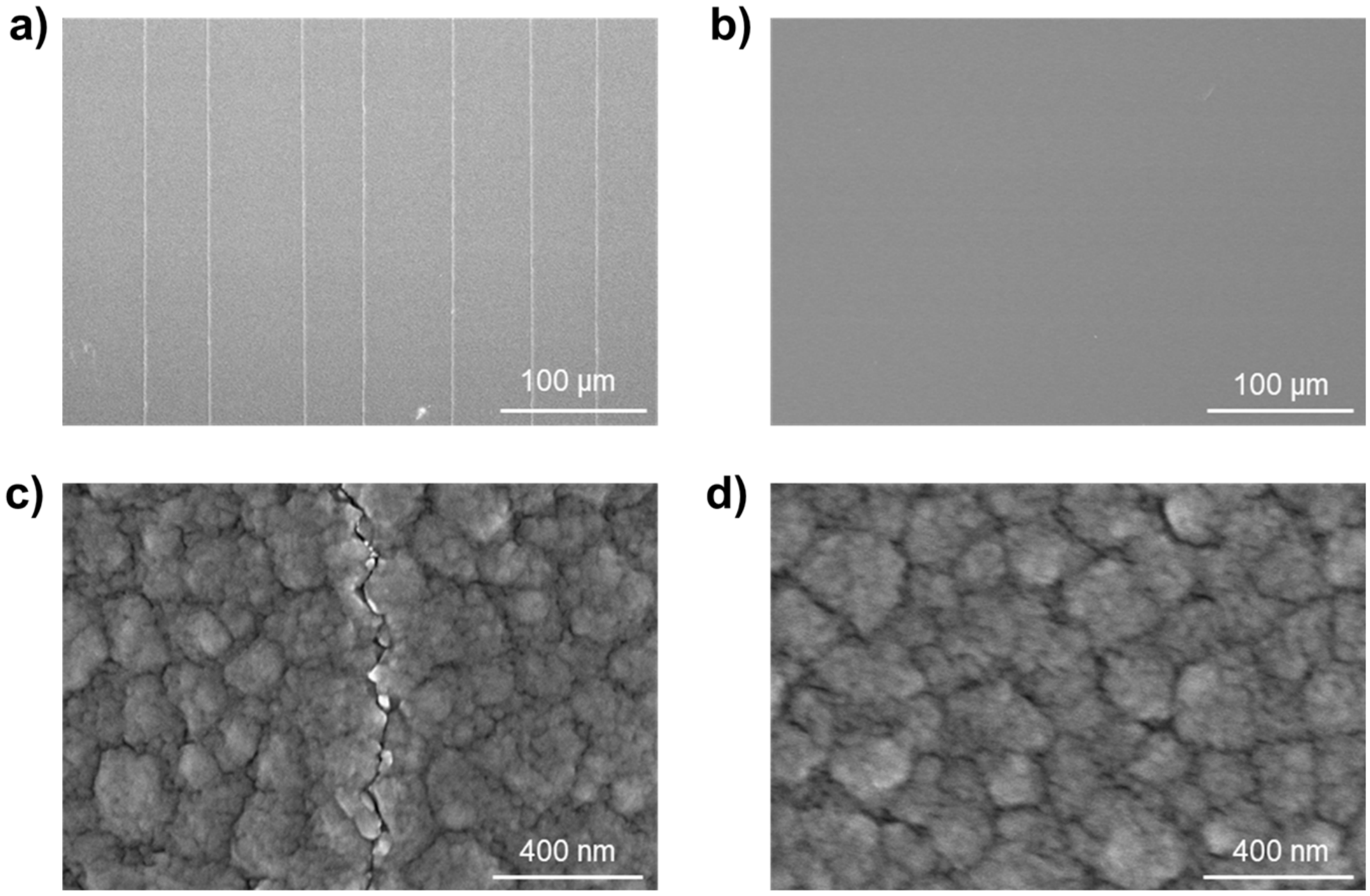

Figure S20: a-b) Low-magnification (×300) and **c-d)** high-magnification (×80K) top-view SEM images of flexible WBG PSCs after bending test under **a, c)** tensile stress and **b, d)** compress stress.

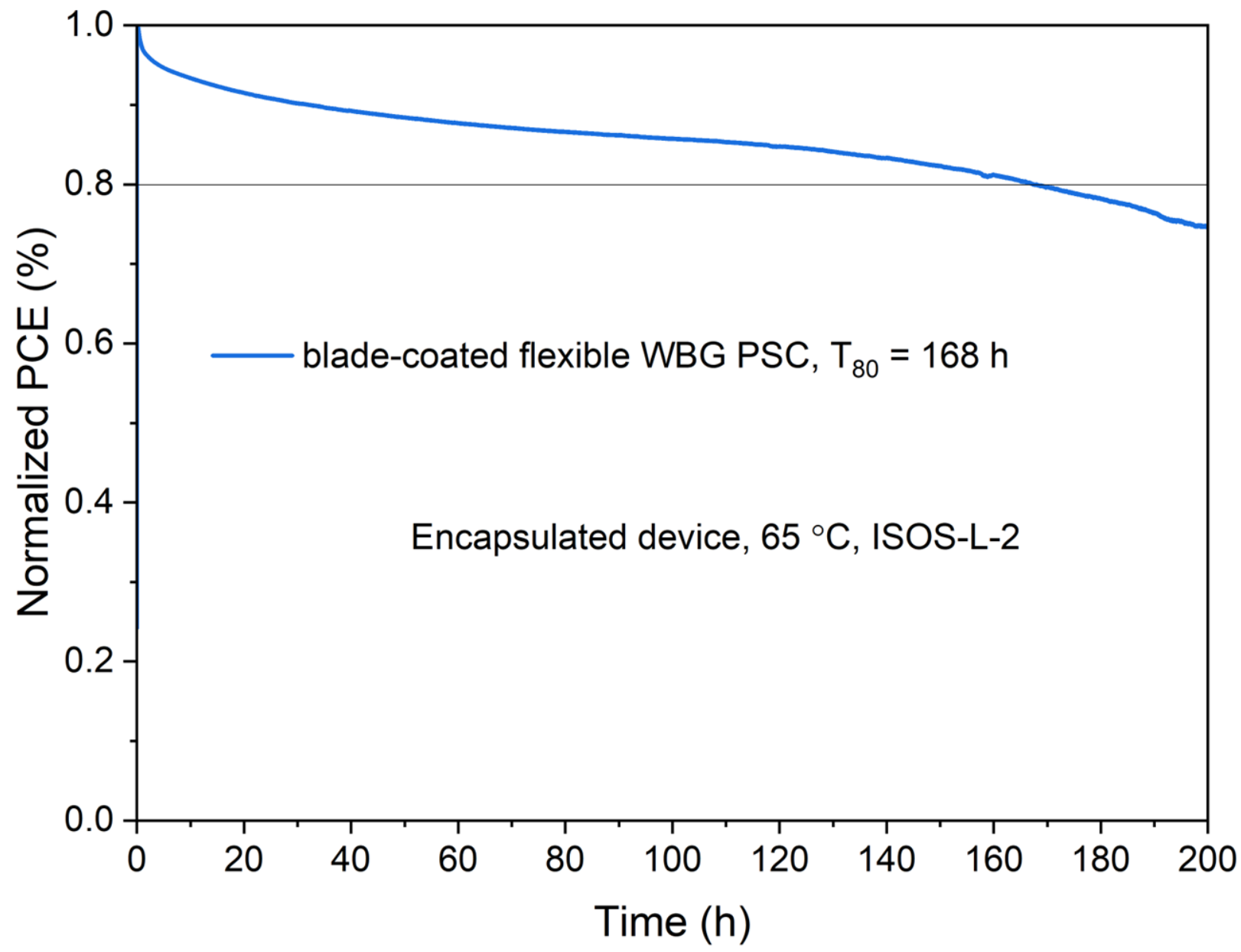

Figure S21: Operational stability of an encapsulated flexible WBG PSC under MPP tracking at 65 °C (ISOS-L-2).

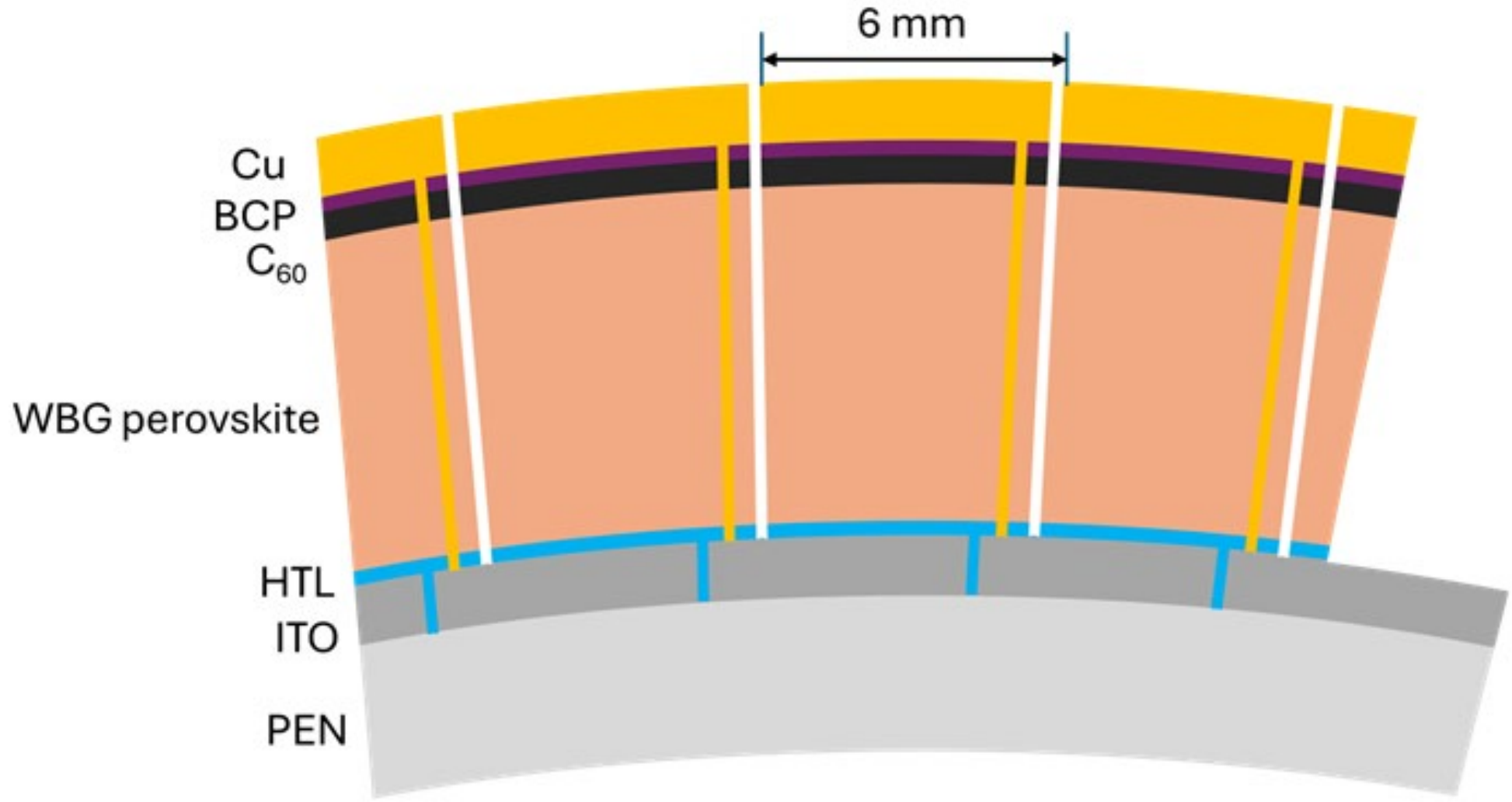

Figure S22: Schematic diagram of a flexible WBG perovskite solar mini-module.

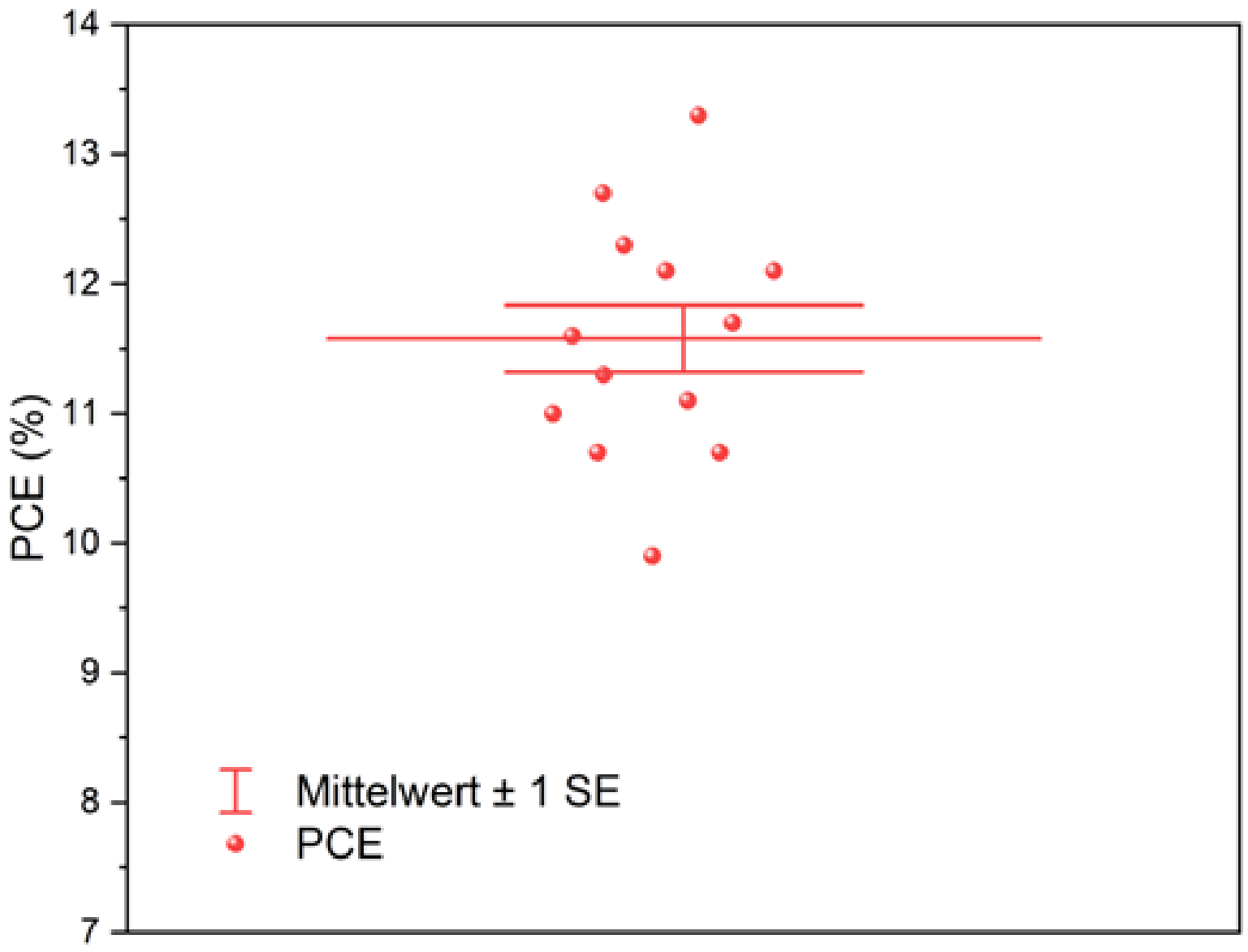

Figure S23: PCE distribution of 13 flexible WBG perovskite solar modules from multiple batches.

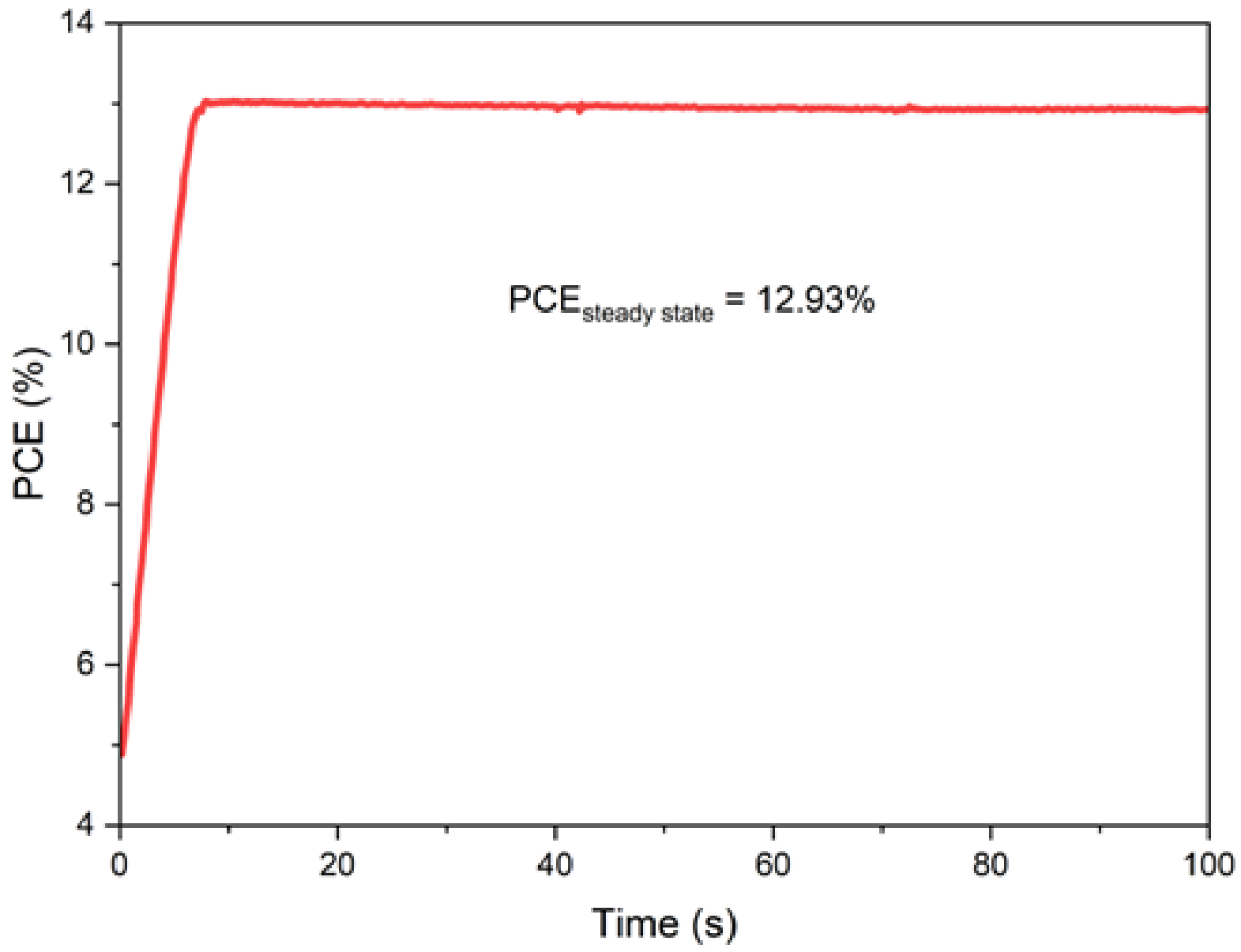

Figure S24: Steady-state efficiency output of the best-performing flexible WBG perovskite solar mini-module.

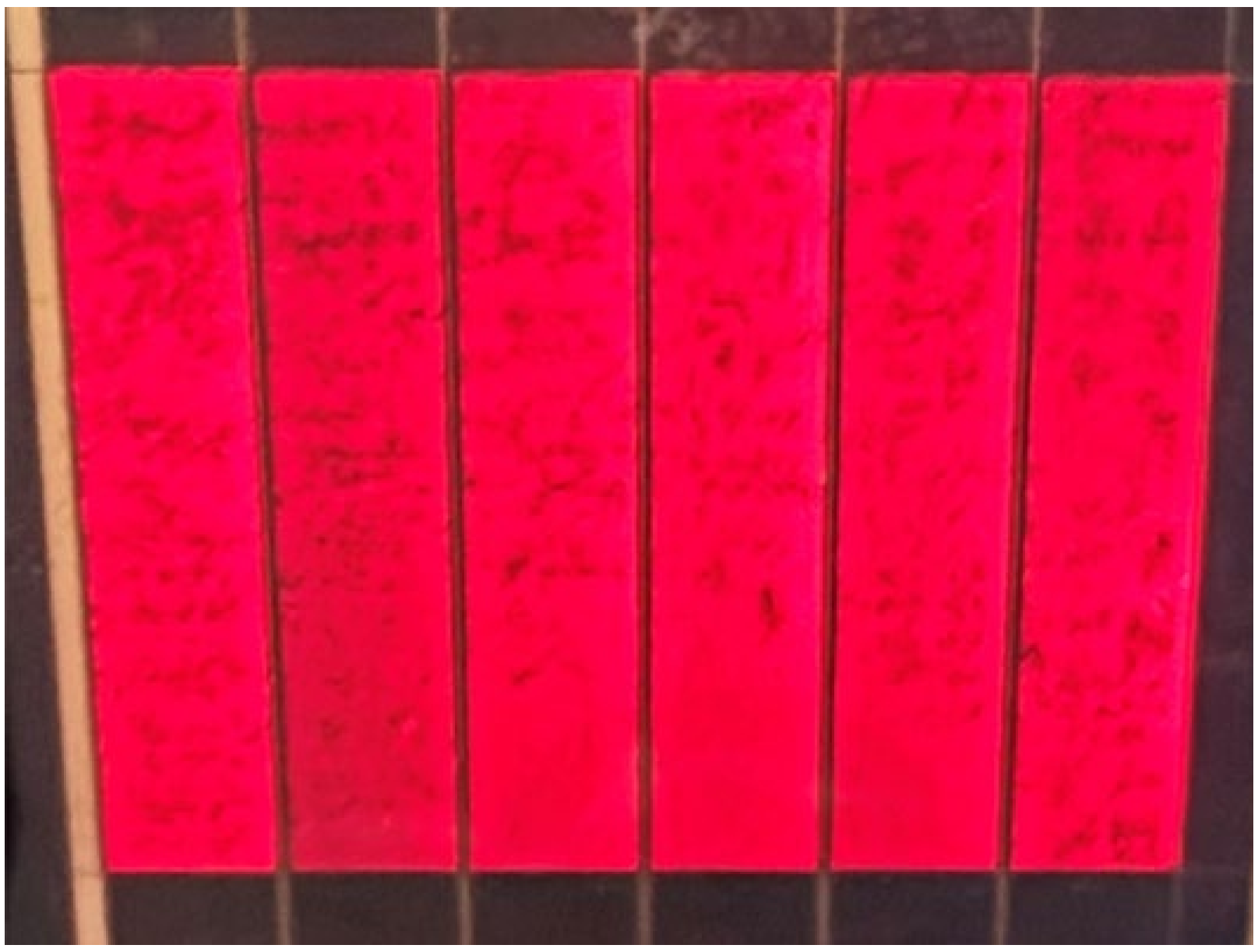

Figure S25: Planar light-emitting image of a flexible WBG perovskite solar mini-module, taken with a mobile phone. The applied voltage is +7.5 V, close to the V_{oc} (7.47 V) of mini-module.

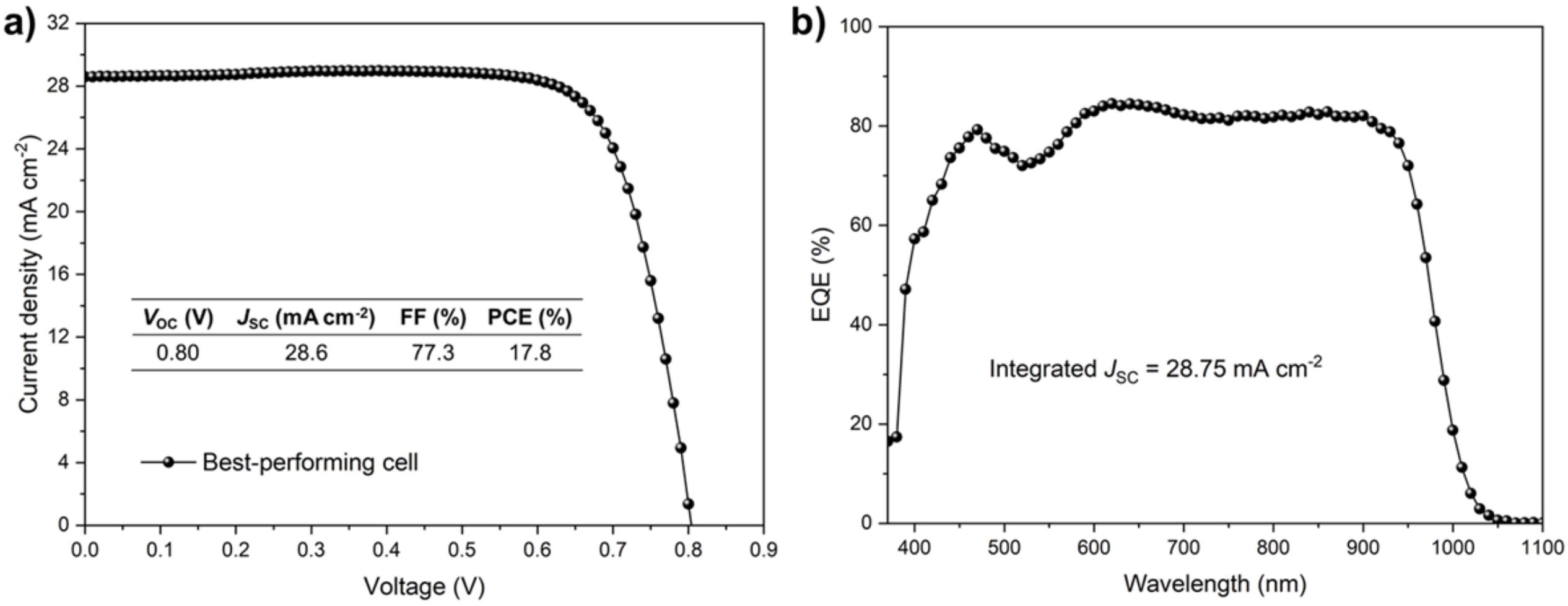

Figure S26: a) *J-V* curve and **b)** EQE spectrum of the flexible NBG perovskite solar cell.

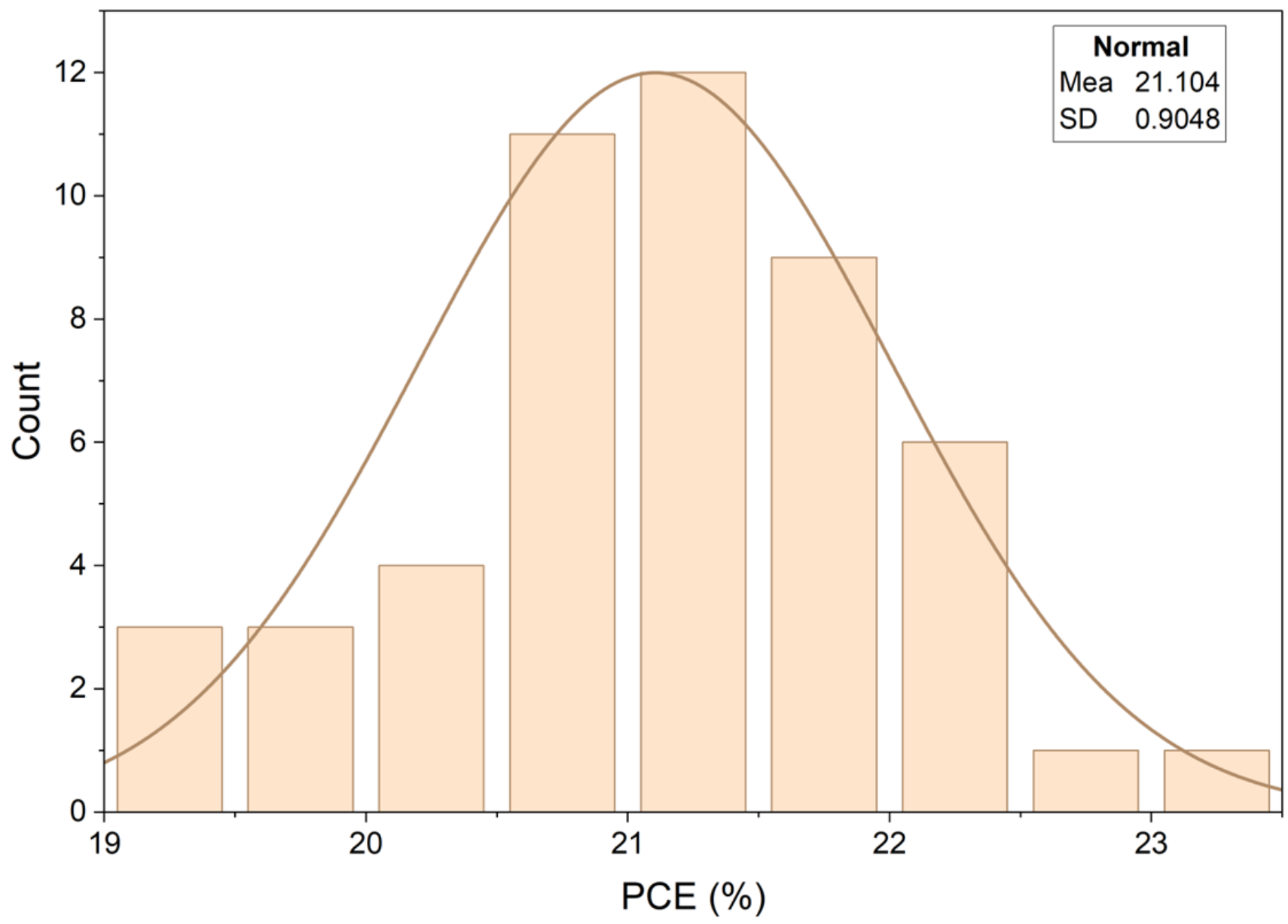

Figure S27: PCE distribution of 50 flexible all-perovskite TSCs from multiple batches.

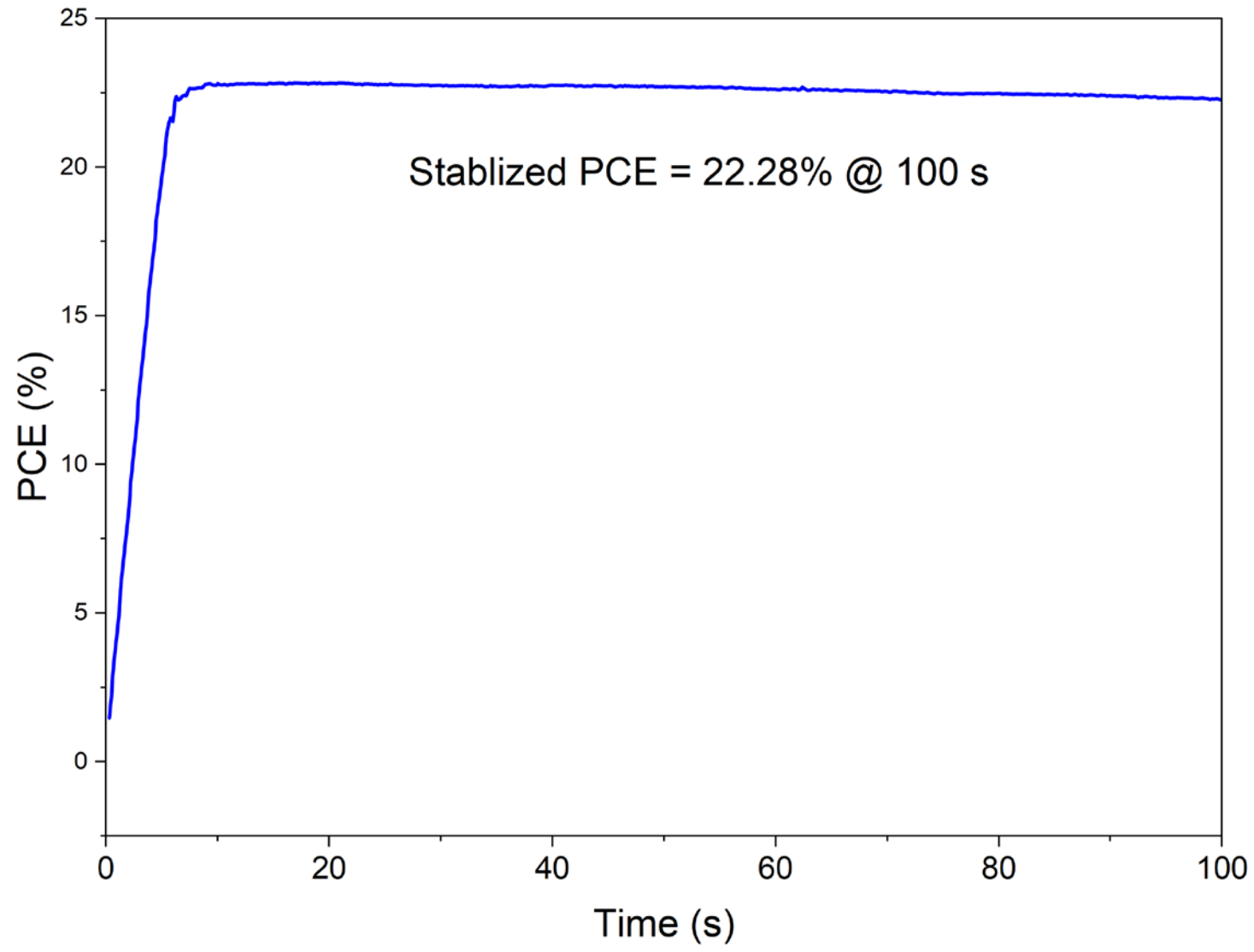

Figure S28: Steady-state efficiency output of the best-performing flexible all-perovskite TSC.

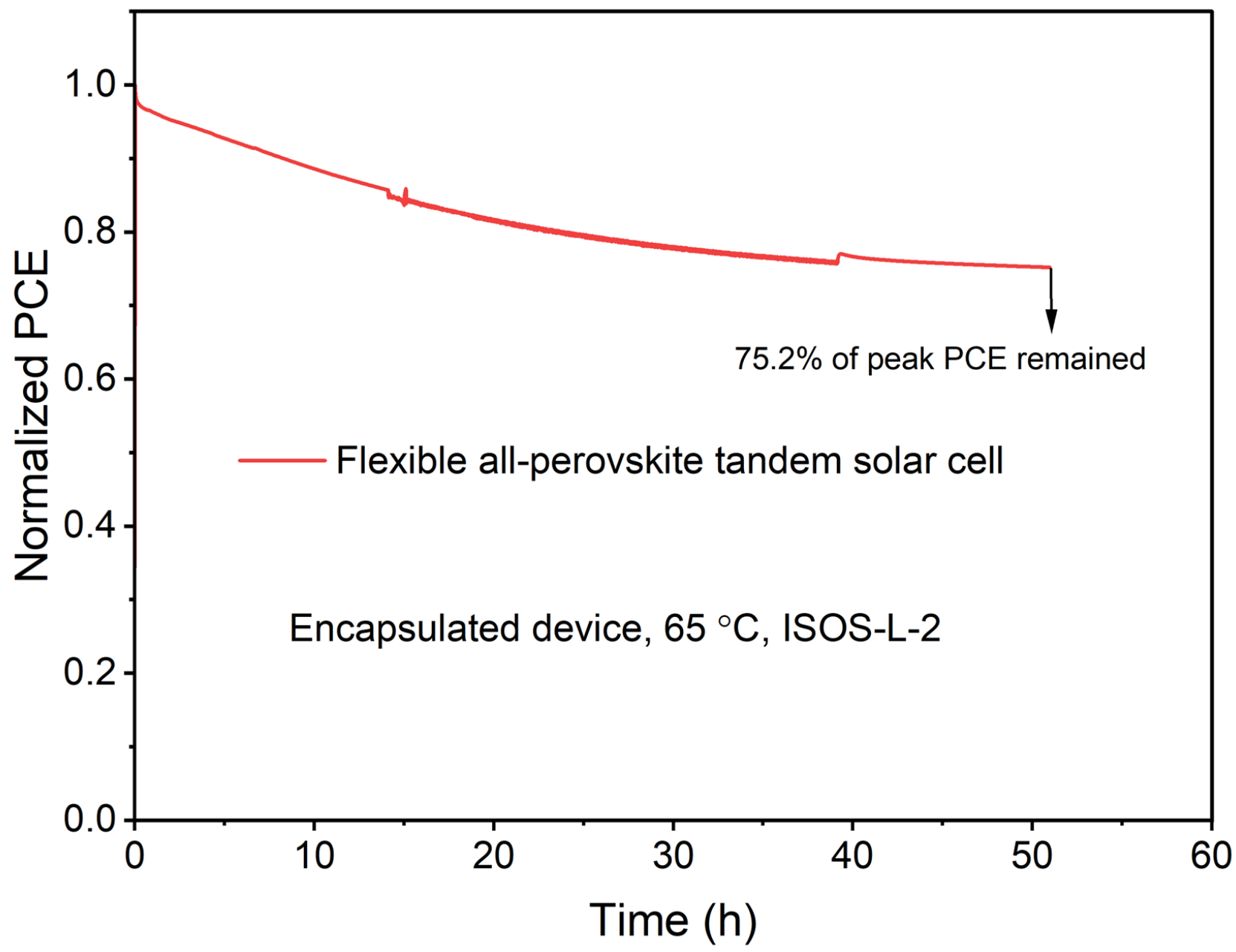

Figure S29: Operational stability of an encapsulated flexible all-perovskite tandem solar cell under MPP tracking at 65 °C (ISOS-L-2).

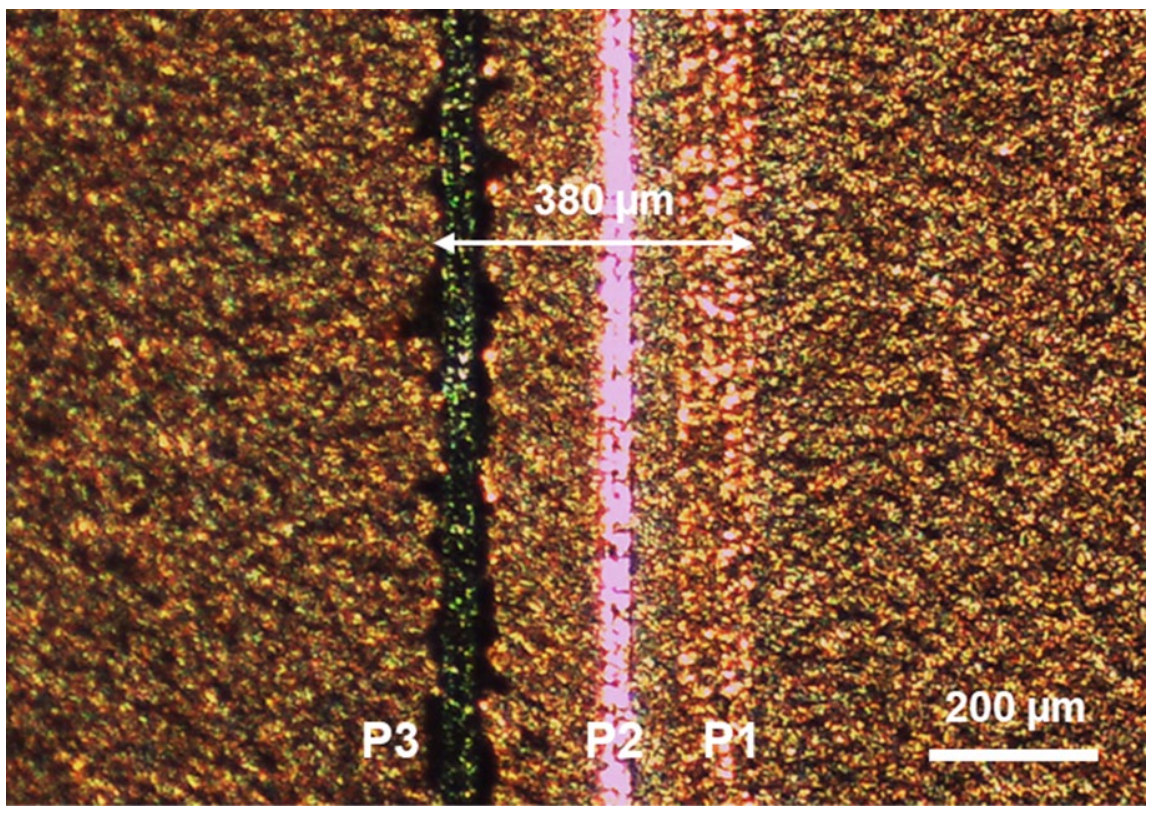

Figure S30: Micrograph of P1-P2-P3 scribe lines of flexible all-perovskite tandem solar mini-module. Scale bar: 200 µm.

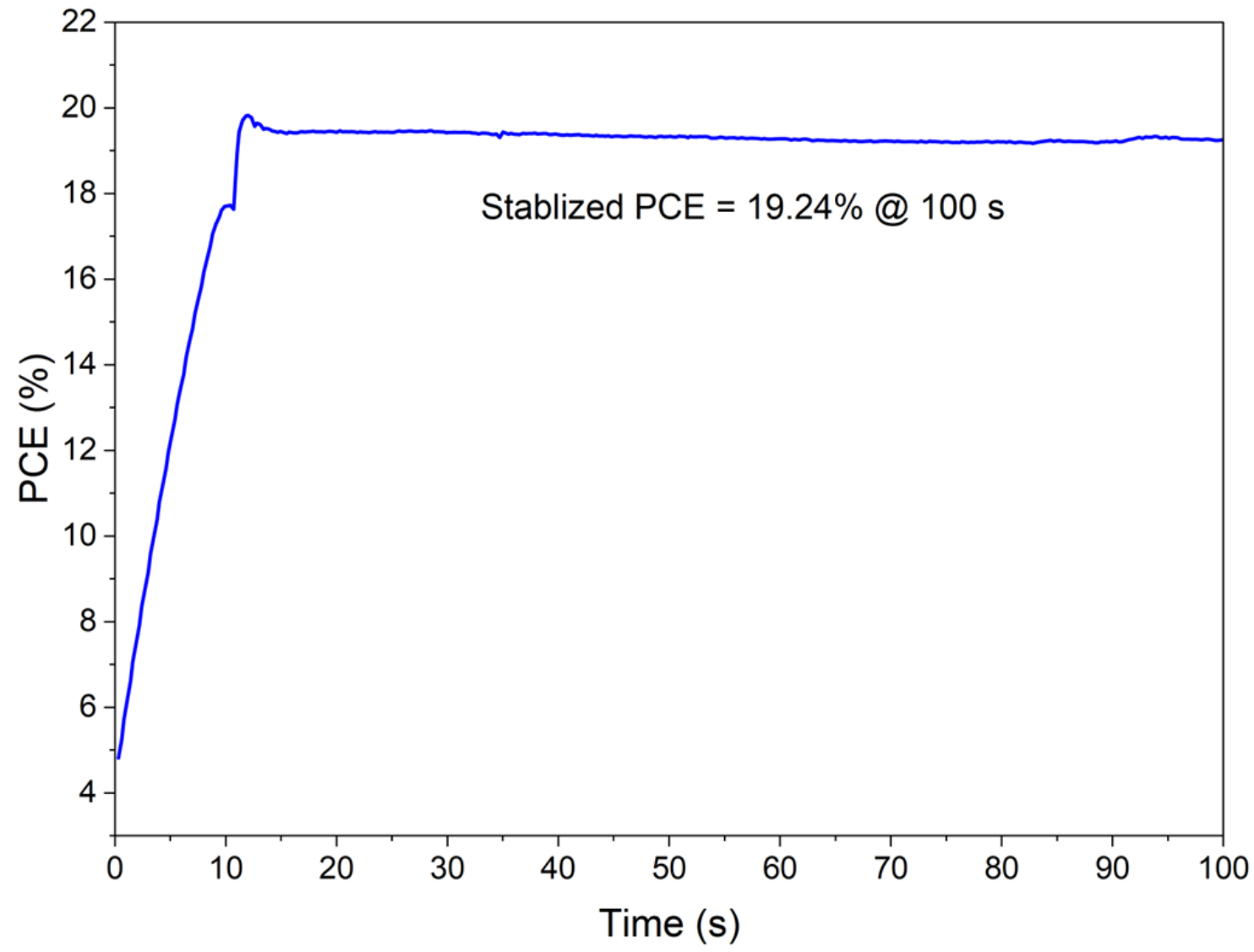

Figure S31: Steady-state efficiency output of the best-performing flexible all-perovskite tandem solar mini-module.

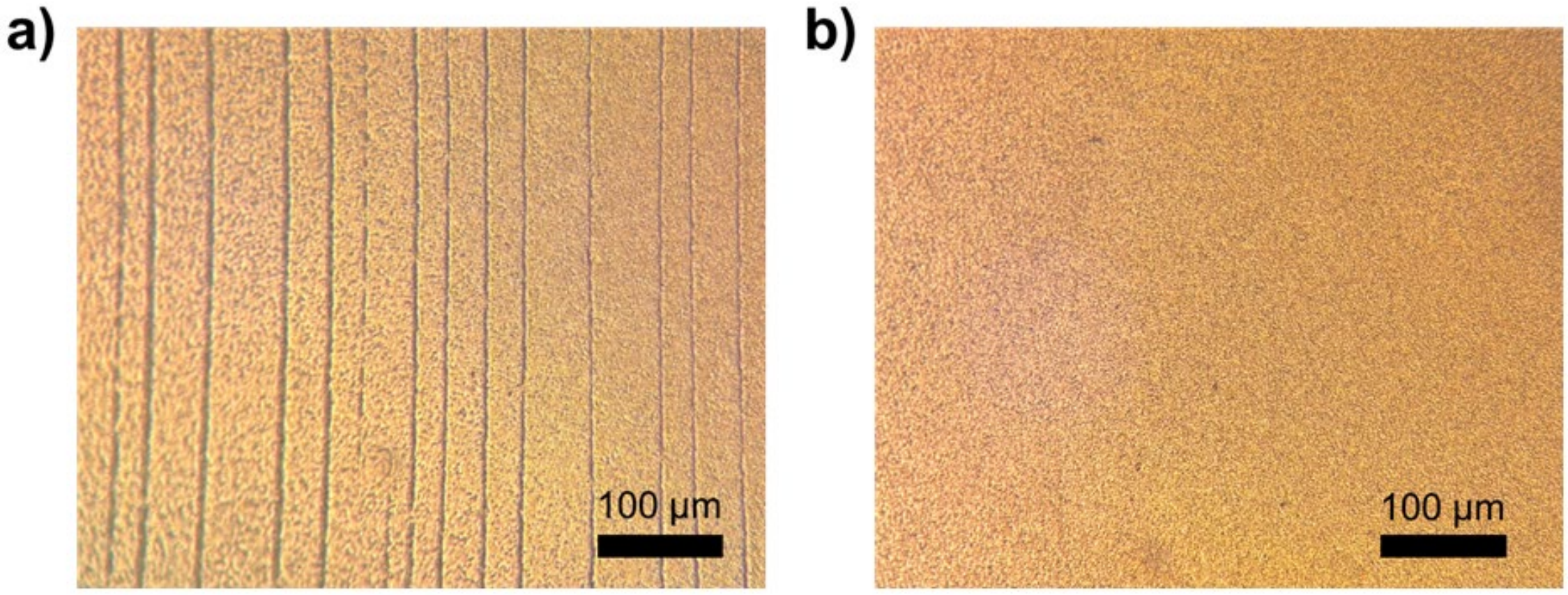

Figure S32: a-b) Micrographs of flexible all-perovskite tandem solar mini-modules after bending tests under tensile stress with minimum bending radius of **a)** 9 mm and **b)** 15 mm. Scale bar: 100 µm.

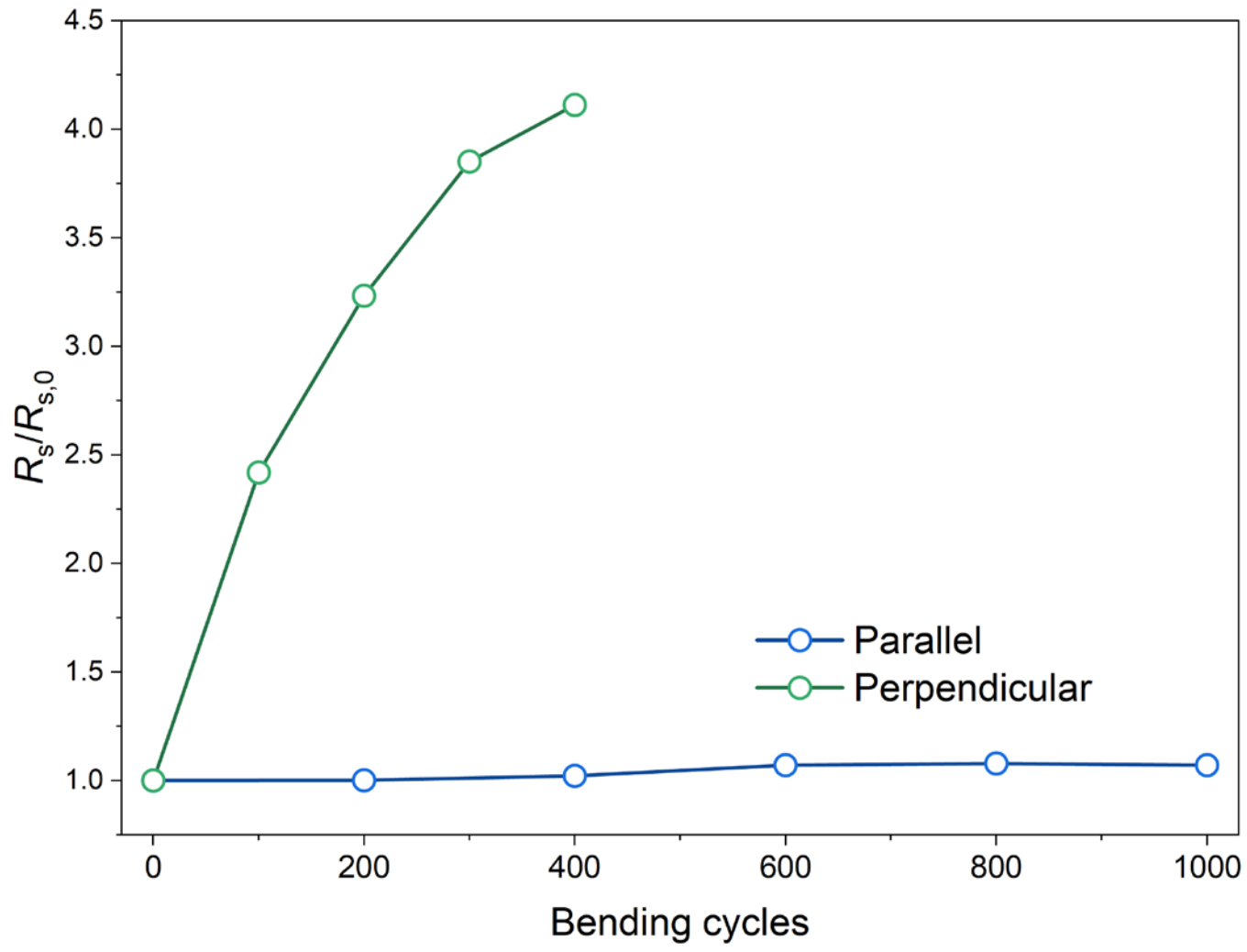

Figure S33: Variation of series resistance (R_s) with different bending cycles under tensile stress, with the bending direction oriented perpendicular or parallel to the scribe lines.

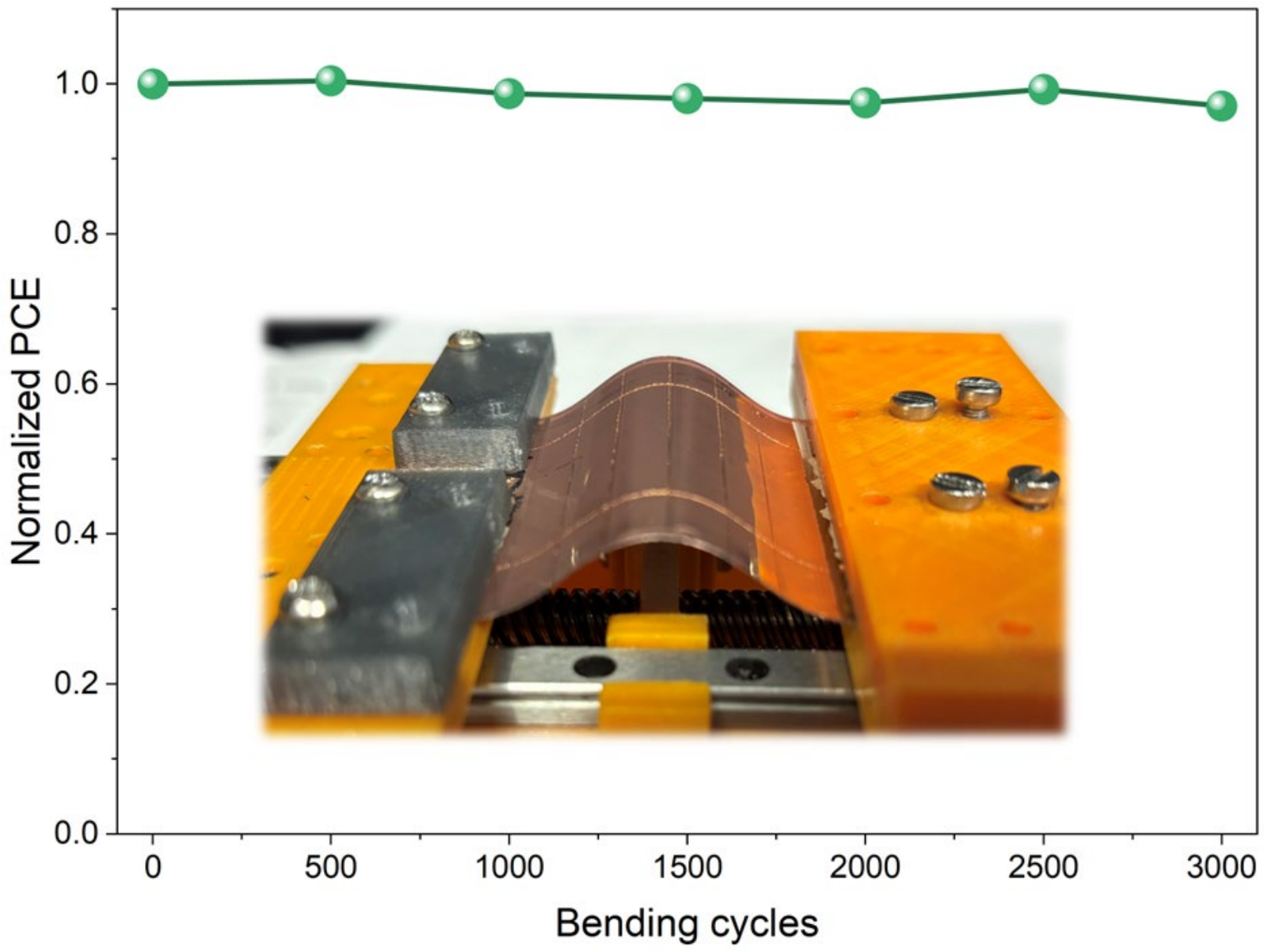

Figure S34: Mechanical bending durability of unencapsulated flexible all-perovskite tandem solar mini-modules under tensile stress at a minimum bending radius of ~15 mm. The inset shows a photograph of a mini-module a curved state during this bending test.

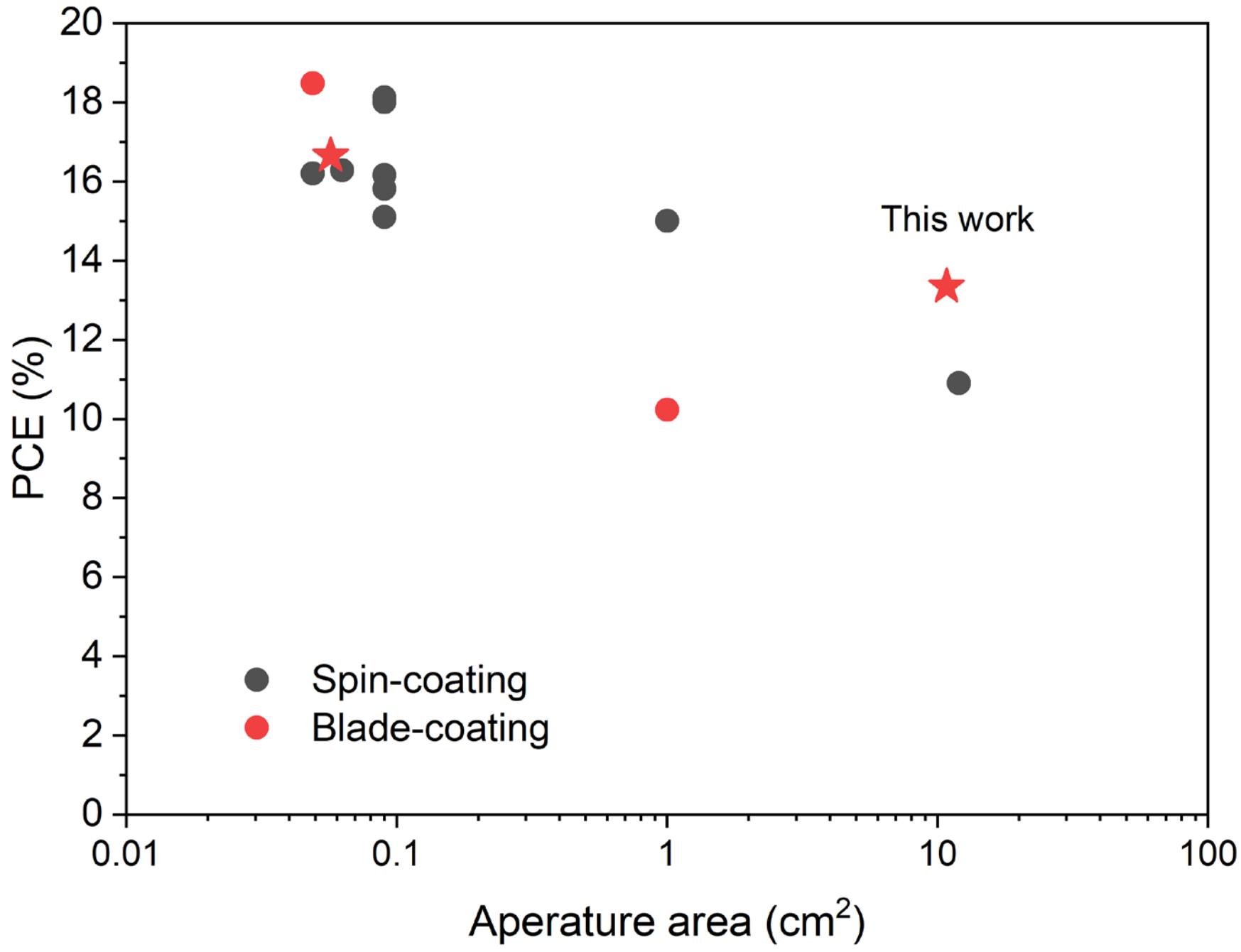

Figure S35: Progress on PCE over the aperture area for flexible WBG perovskite solar cells and minimodules. More detailed information is listed in **Table S2**.

Note S3: Scalable deposition of NiO_x over large-area flexible substrates

The spatial uniformity of spin-coated NiO_x films on large-area substrates is significantly limited. During spin coating, film formation is governed by centrifugal spreading, solvent evaporation, and edge-bead formation, which can lead to thickness gradients and poor reproducibility when the substrate size increases. These limitations become even more pronounced on flexible substrates because of their surface unevenness. Therefore, developing scalable deposition techniques is highly desirable for industry-compatible production of flexible perovskite solar modules.

Sputtering and slot-die coating are two promising approaches for depositing uniform NiO_x films over large-area substrates. Sputtering offers good thickness control, dense film formation, and compatibility with established thin-film photovoltaic manufacturing lines. However, for flexible perovskite photovoltaics, sputtering may introduce several practical concerns, like sputtering-induced damage to underlying layers, and limit production throughput. By contrast, slot-die coating is a solution-processed, roll-to-roll-compatible technique that enables continuous, material-efficient, and large-area deposition under relatively mild processing conditions. Its thickness can be controlled by ink concentration, flow rate, coating speed, and coating gap. Therefore, compared with sputtering, slot-die coating represents a more suitable and industry-compatible strategy for scalable NiO_x deposition over large-area flexible substrates.[7]

Table S1: Bi-exponential fitting results of TRPL decay curves shown in **Figure S9**.

	τ_1 (ns)	τ_2 (ns)
NiO_x/Me-4PACz	70.46	385.26
NiO_x/4PADCB	79.88	393.73
NiO_x/2PACz	118.32	395.05
NiOx/MeO-2PACz	65.14	269.21

Table S2: A summary of flexible WBG (~1.8 eV) perovskite solar cells and mini-modules.

Publication year	Bandgap (eV)	PCE (%)	Area (cm^2)	Coating method	Ref
2022	1.77	15.1	0.09	spin-coating	[8]
2022	1.75	16.2	0.049	spin-coating	[6]
2024	1.8	16.16	0.09	spin-coating	[9]
2025	1.76	15	1	spin-coating	[10]
2025	1.83	16.28	0.0628	spin-coating	[11]
2026	1.8	15.81	0.09	spin-coating	[12]
2026	1.83	18	0.09	spin-coating	[13]
2026	1.83	18.13	0.09	spin-coating	[14]
2026	1.73	10.9	12	spin-coating	[15]
2022	1.7	10.23	1	blade-coating	[16]
2025	1.8	18.48	0.049	blade-coating	[17]
**This work**	**1.8**	**16.65**	**0.057**	**blade-coating**	
		**13.34**	**10.8**	**blade-coating**	

References

[1] S. Sascha, G. Thilo, *Springer Series in Surface Sciences* **2018**, 65. https://doi.org/10.1007/978-3-319-75687-5_1

[2] J. W. Kim, A. Kim, H. U. Hwang, J. H. Kim, S. Choi, N. Koch, D. Shin, Z. Zhao, F. Liu, M. Choi, K. M. Lee, Y. Park, *Journal of Vacuum Science & Technology A* **2023**, 41. https://doi.org/10.1116/6.0002852

[3] Vitios solar cell imaging tool, Fluxim AG, Switzerland, https://www.fluxim.com/vitios,

[4] T. Kirchartz, J. A. Márquez, M. Stolterfoht, T. Unold, *Advanced Energy Materials* **2020**, 10. https://doi.org/10.1002/aenm.201904134

[5] A. Al-Ashouri, E. Kohnen, B. Li, A. Magomedov, H. Hempel, P. Caprioglio, J. A. Marquez, A. B. Morales Vilches, E. Kasparavicius, J. A. Smith, N. Phung, D. Menzel, M. Grischek, L. Kegelmann, D. Skroblin, C. Gollwitzer, T. Malinauskas, M. Jost, G. Matic, B. Rech, R. Schlatmann, M. Topic, L. Korte, A. Abate, B. Stannowski, D. Neher, M. Stolterfoht, T. Unold, V. Getautis, S. Albrecht, *Science* **2020**, 370, 1300. https://doi.org/10.1126/science.abd4016

[6] L. Li, Y. Wang, X. Wang, R. Lin, X. Luo, Z. Liu, K. Zhou, S. Xiong, Q. Bao, G. Chen, Y. Tian, Y. Deng, K. Xiao, J. Wu, M. I. Saidaminov, H. Lin, C.-Q. Ma, Z. Zhao, Y. Wu, L. Zhang, H. Tan, *Nature Energy* **2022**, 7, 708. https://doi.org/10.1038/s41560-022-01045-2

[7] Y. Zhu, Y. Zhang, M. Hu, Y. Hao, W. Huang, H. Park, J. Lu, *Advanced Functional Materials* **2025**, 36. https://doi.org/10.1002/adfm.202512921

[8] H. Lai, J. Luo, Y. Zwirner, S. Olthof, A. Wieczorek, F. Ye, Q. Jeangros, X. Yin, F. Akhundova, T. Ma, R. He, R. K. Kothandaraman, X. Chin, E. Gilshtein, A. Müller, C. Wang, J. Thiesbrummel, S. Siol, J. M. Prieto, T. Unold, M. Stolterfoht, C. Chen, A. N. Tiwari, D. Zhao, F. Fu, *Advanced Energy Materials* **2022**, 12. https://doi.org/10.1002/aenm.202202438

[9] Z. Xie, S. Chen, S. Zhang, Y. Pei, L. Li, P. Wu, *Small* **2025**, 21, e2410022. https://doi.org/10.1002/smll.202410022

[10] A. Wąsiak-Maciejak, Ł. Przypis, W. Żuraw, K. Rycek, P. Janicka, M. Ścigaj, K. Dyk, H. Lai, A. Piejko, D. Pucicki, F. Fu, V. Kinzhybalo, K. Wojciechowski, *Journal of Materials Chemistry A* **2025**, 13, 7335. https://doi.org/10.1039/d4ta07266a

[11] J. Cao, W. Chen, C. Zhao, J. Xu, J. Zheng, S. Kang, J. Zhu, J. Zhang, Y. Li, *Science China Materials* **2025**, 68, 1415. https://doi.org/10.1007/s40843-024-3209-x

[12] Z. Xie, S. Zhang, S. Chen, Y. Pei, L. Li, J. Yang, G. Fu, P. Wu, *Solar Energy Materials and Solar Cells* **2026**, 295. https://doi.org/10.1016/j.solmat.2025.113993

[13] R. Jiang, Y. Yun, K. Sun, X. Cui, Q. Zhang, C. Hu, C. Jin, Q. Kong, Y. Liu, J. Gong, S. He, L. Qiu, C. Liu, S. Bai, C. Li, Z. Bo, F. Huang, Y. B. Cheng, T. Bu, *Nat Commun* **2026**, 17, 1375. https://doi.org/10.1038/s41467-025-68125-1

[14] Q. Zhang, R. Jiang, Y. Yun, X. Zhu, K. Sun, C. Hu, J. Wu, Z. Shi, J. Gao, J. Gong, S. Bai, F. Huang, Y. B. Cheng, T. Bu, *InfoMat* **2026**, 8. https://doi.org/10.1002/inf2.70132

[15] A. Labiod, P. Tsoulka, C. Leon, J. Allegre, S. Berson, *Advanced Photonics Research* **2026**, 7. https://doi.org/10.1002/adpr.202500286

[16] V. Babu, M. A. Mejia Escobar, R. Fuentes Pineda, M. Ścigaj, P. Spinelli, K. Wojciechowski, *Materials Today Energy* **2022**, 28. https://doi.org/10.1016/j.mtener.2022.101073

[17] M. Li, H. Gao, L. Li, E. Wang, Z. Liu, I. T. Cheong, P. Wu, Y. Zhang, Y. Wang, X. Zheng, M. Yin, R. Lin, R. Liu, H. Luo, K. Xiao, W. Kong, W. Sun, Y. Nie, X. Luo, M. I. Saidaminov, Y. Li, H. Tan, *Nature Photonics* **2025**, 19, 1255. https://doi.org/10.1038/s41566-025-01746-6
